# Magnetohydrodynamic Flow by a Stretching Cylinder with Newtonian Heating and Homogeneous-Heterogeneous Reactions

**DOI:** 10.1371/journal.pone.0156955

**Published:** 2016-06-09

**Authors:** T. Hayat, Zakir Hussain, A. Alsaedi, M. Farooq

**Affiliations:** 1 Department of Mathematics, Quaid-I-Azam University 45320, Islamabad 44000, Pakistan; 2 Nonlinear Analysis and Applied Mathematics (NAAM) Research Group, Department of Mathematics, Faculty of Science, King Abdulaziz University, P. O. Box 80257, Jeddah 21589, Saudi Arabia; 3 Department of Mathematics, Riphah International University, Islamabad 44000, Pakistan; IUMPA - Universitat Politecnica de Valencia, SPAIN

## Abstract

This article examines the effects of homogeneous-heterogeneous reactions and Newtonian heating in magnetohydrodynamic (MHD) flow of Powell-Eyring fluid by a stretching cylinder. The nonlinear partial differential equations of momentum, energy and concentration are reduced to the nonlinear ordinary differential equations. Convergent solutions of momentum, energy and reaction equations are developed by using homotopy analysis method (HAM). This method is very efficient for development of series solutions of highly nonlinear differential equations. It does not depend on any small or large parameter like the other methods i. e., perturbation method, *δ*—perturbation expansion method etc. We get more accurate result as we increase the order of approximations. Effects of different parameters on the velocity, temperature and concentration distributions are sketched and discussed. Comparison of present study with the previous published work is also made in the limiting sense. Numerical values of skin friction coefficient and Nusselt number are also computed and analyzed. It is noticed that the flow accelerates for large values of Powell-Eyring fluid parameter. Further temperature profile decreases and concentration profile increases when Powell-Eyring fluid parameter enhances. Concentration distribution is decreasing function of homogeneous reaction parameter while opposite influence of heterogeneous reaction parameter appears.

## 1 Introduction

Non-Newtonian fluids play key role in various industrial processes such as natural products, multiphase mixtures, biological fluids, food products, agricultural and daily food wastes. Due to its highly utility and wide range of applications in industries researchers have keen interest to explore the characteristics of non-Newtonian fluids. The non-Newtonian fluids have nonlinear relationship between stress and rate of strain. Such fluids cannot be predicted by a single constitutive equation due to their diverse characteristics. One of the non-Newtonian fluids is the Powell-Eyring fluid which was proposed by Powell and Eyring in 1944. It has several advantages i.e., (i) It is derived from kinetic theory of liquid rather than the empirical relation. (ii) At low and high shear stresses Powell-Eyring fluid behaves as a Newtonian. The flow of the non-Newtonian fluids plays significant role in various industrial, natural, engineering and geophysical processes. These processes involve manufacturing of cooling system with liquid metals, MHD generators, accelerators, nuclear reactors, electric motors, blood flow measurements, pumps and flow meters etc. MHD flow through blood vessel has gained considerable attention in physiological processes. Due to such considerable applications scientists and researchers carried out magnetohydrodynamic flows regarding different physical phenomenon. Hayat et al. [1] analyzed melting heat transfer in the stagnation point flow of Powell–Eyring fluid. Ellahi et al. [2] developed numerical analysis for MHD steady flow with heat transfer and nonlinear slip effects. Javed et al. [3] discussed Powell-Eyring fluid flow over a stretching sheet. Ellahi [4] studied the influence of temperature dependent viscosity on the MHD flow of non-Newtonian nanofluid in a pipe. Hayat et al. [5] examined radiative effects in three-dimensional flow of MHD Eyring-Powel fluid. Zeeshan et al. [6] analyzed the magnetohydrodynamic flow of non-Newtonian fluid in porous space with slip boundary conditions. Ara et al. [7] studied radiation effect on boundary layer flow of Powell-Eyring fluid by an exponentially shrinking sheet. Magnetohydrodynamic flow of water/ethylene glycol based nanofluids with natural convection and porous medium is addressed by Zeeshan et al. [8]. Steady flow of an Eyring Powell fluid over a moving surface with convective boundary conditions was presented by Hayat et al. [9]. Ellahi et al. [10] presented the MHD blood flow of Prandtl fluid between permeable walls through tapered stenosed arteries. Study of stream wise transverse magnetic fluid flow with heat transfer by porous obstacle studied by Rashidi et al. [11].

In current time the development of human society greatly depends on the energy resources. Researchers and scientists have stimulated in this area to develop advance energy resources and technologies so that solar energy could be utilized in a more easy and efficient way. In this regards it is desired to design and construct high rate of heating/cooling equipment machinery and various medical instruments and devices with high capability of chemical transport. Heat transfer in various natural and industrial process depend upon mechanism of heat transfer from wall to ambient fluid. Merkin [12] suggested four common ways of heat transfer from wall to ambient temperature distribution in 1994 i.e., constant or prescribed surface temperature, constant or prescribe surface flux, conjugate or convective boundary condition and Newtonian heating where the heat transfer from any material surface with a finite heat capacity is proportional to the local surface temperature. Newtonian heating phenomenon is especially important in practical applications such as to design heat exchanger, conjugate heat transfer around fins and also in convective flow. Hayat et al. [13] explored stagnation point flow of Burgers fluid with Newtonian heating. Salleh et al. [14–15] discussed in detail about free convection boundary layer flow of micropolar fluid due to solid sphere with Newtonian heating.

Scientists and researchers are desired to develop and design some new catalytic processes which work at very high temperature. In these processes homogeneous and heterogeneous chemical reactions play significant role. Homogeneous and heterogeneous reactions are very complex involving consumption and production of reactant species both with in the fluid and on the catalyst surface at different rates. Such reactions involve in combustion, catalysis and bio chemical systems. Merkin [16] discussed the isothermal model for homogeneous heterogeneous reactions in the boundary layer flow. Shaw et al. [17] investigated the effects of homogeneous-heterogeneous reactions in the micropolar fluid induced by stretching/shrinking sheet embedded in a porous medium. Kameswaran et al. [18] discussed the analysis of homogeneous-heterogeneous reactions in flow of nanofluid past a permeable stretching sheet. Kumar et al. [19] investigated the effects of homogeneous and heterogeneous reactions on the dispersion of a solute for immiscible viscous fluids between two plates. Hayat et al. [20] discussed homogeneous-heterogeneous reactions in the stagnation point flow of carbon nanotubes with Newtonian heating.

The main objective here is to disclose the characteristics of homogeneous-heterogeneous reactions in the MHD flow of Powell-Eyring fluid over an impermeable stretching cylinder. Heat transfer analysis is carried out with Newtonian heating and heat generation/absorption. Diffusion coefficients of both species are considered to be equal. Homotopy analysis method [21–29] is used to achieve the convergent series solutions of momentum, energy and concentration equations. Behaviors of various pertinent parameters on the velocity, temperature and concentration distributions are analyzed through graphs. Present results are compared with the previous published data in the limiting case.

## 2 Mathematical formulation

Consider the MHD two-dimensional boundary layer flow of Powell-Eyring fluid by a stretching cylinder. Heat transfer includes the salient features of heat generation/absorption. Uniform magnetic field *β*_0_ is applied in the radial direction. Effect of induced magnetic field due to small magnetic Reynolds number is neglected. Further the effects of homogeneous- heterogeneous reactions and Newtonian heating are also considered. The heat produced is ignored during the irreversible chemical reaction. The homogeneous reaction for cubic autocatalysis can be expressed in following fashion:

$$\begin{array}{c} A+2B\to 3B,rate=k_{1}ab^{2}, \end{array}$$ (1)

while first-order isothermal reaction on the catalyst surface is given by

$$\begin{array}{c} A\to B,rate=k_{s}a. \end{array}$$ (2)

**Fig 1:**
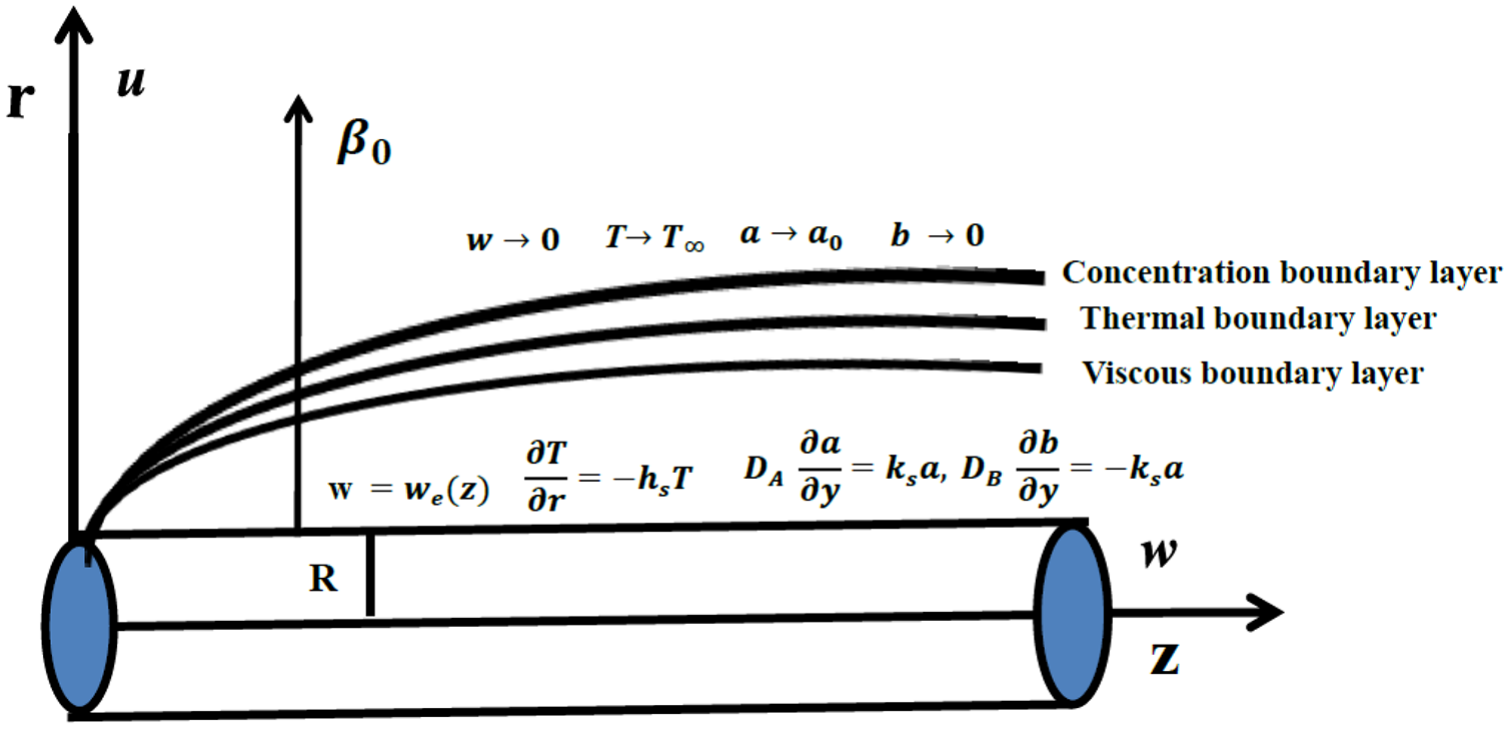
Geometry of problem.

Here the concentrations of chemical species **A** and **B** are denoted by *a* and *b* while k_1_ and k_*s*_ are the rate constants. This irreversible reaction assures that the reaction rate is negligible in the external flow and at the outer edge of the boundary layer. Cylindrical coordinates are chosen such that *z*—axis is along the stretching cylinder and *r*—axis normal to it (See Fig 1). The extra stress tensor of Powell-Eyring model is

$$\begin{array}{c} \tau =\left\{\mu +\frac{1}{\beta \zeta^{\cdot }}\sinh^{-1}\left(\frac{1}{c}\zeta^{\cdot }\right)\right\}A_{1}, \end{array}$$ (3)

$$\begin{array}{c} \zeta^{\cdot }=\sqrt{\frac{1}{2}tr{\left(A_{1}\right)}^{2}}, \end{array}$$ (4)

where *μ* denotes the dynamic viscosity of fluid, *β* and *c* are the fluid parameters of the Powell-Eyring model and c has the dimension of (*time*)^−1^. Here second-order approximation of the sinh^−1^ function is considered

$$\begin{array}{c} \sinh^{-1}\left(\frac{1}{c}\zeta^{\cdot }\right)≅\frac{\zeta^{\cdot }}{c}-\frac{\zeta^{\cdot 3}}{6c^{3}}, \end{array}$$ (5)

with

$$\begin{array}{c} \frac{\zeta^{\cdot 5}}{c^{5}}<<<1. \end{array}$$

Under the boundary layer approximations (i.e, *u* = *O*(*δ*), *r* = *O*(*δ*), *w* = *O*(1) and *z* = *O*(1)) the continuity, momentum, energy and homogeneous-heterogeneous reactions equations are as follows:

$$\frac{\partial \left(ru\right)}{\partial r}+\frac{\partial \left(rw\right)}{\partial z}=0,$$ (6)

$$\begin{array}{ll} u\frac{\partial w}{\partial r}+w\frac{\partial w}{\partial z} & =\nu \left(\frac{\partial^{2}w}{\partial r^{2}}+\frac{1}{r}\frac{\partial w}{\partial r}\right)+\frac{1}{\rho \beta c}\left(\frac{\partial^{2}w}{\partial r^{2}}+\frac{1}{r}\frac{\partial w}{\partial r}\right)\\ & -\frac{1}{6\rho \beta c^{3}}\left(\frac{1}{r}{\left(\frac{\partial w}{\partial r}\right)}^{3}+3{\left(\frac{\partial w}{\partial r}\right)}^{2}\left(\frac{\partial^{2}w}{\partial r^{2}}\right)\right)-\frac{\sigma_{1}\beta_{0}^{2}}{\rho }u, \end{array}$$ (7)

$$u\frac{\partial T}{\partial r}+w\frac{\partial T}{\partial z}=\frac{k}{\rho c_{p}}\left(\frac{\partial^{2}T}{\partial r^{2}}+\frac{1}{r}\frac{\partial T}{\partial r}\right)+\frac{Q}{\rho c_{p}}\left(T-T_{\infty }\right),$$ (8)

$$\begin{array}{l} u\frac{\partial a}{\partial r}+w\frac{\partial a}{\partial z}=D_{A}\left(\frac{\partial^{2}a}{\partial r^{2}}+\frac{1}{r}\frac{\partial a}{\partial r}\right)-k_{1}ab^{2},\\ u\frac{\partial b}{\partial r}+w\frac{\partial b}{\partial z}=D_{B}\left(\frac{\partial^{2}b}{\partial r^{2}}+\frac{1}{r}\frac{\partial b}{\partial r}\right)+k_{1}ab^{2}. \end{array}$$ (9)

The boundary conditions are prescribed into the forms [16]:

$$\begin{array}{c} w=w_{e}=\frac{U_{0}z}{l},\;\;\;u=0,\;\;\;\frac{\partial T}{\partial r}\;=-h_{s}\;T\;\text{,}\\ D_{A}\frac{\partial a}{\partial r}=k_{s}a,\;\;\;D_{B}\frac{\partial b}{\partial r}=-k_{s}a\;\;\;\;\;\text{at}\;r=R\;\text{,}\\ w\to 0,\;\;\;T\to T_{\infty },\;\;a\to a_{0},\;\;\;b\to 0\;\;\;\;\text{as}\;r\to \infty , \end{array}$$ (10)

where *u* and *w* denote the velocity components in the *r*− and *z*− directions respectively, *U*_0_ is the reference velocity, *l* is the characteristics length, *ν* is the kinematic viscosity, *ρ* is the density, *c*_*p*_ is the specific heat, *k* is the thermal conductivity, *T* and *T*_∞_ are the temperatures of fluid and ambient respectively, *β*_0_ is strength of applied magnetic field, *σ*_1_ is electrical conductivity, *Q* is the coefficient of heat generated or absorbed per unit volume, *w*_*e*_ is the stretching velocity, *h*_*s*_ is the heat transfer coefficient, *D*_*A*_ and *D*_*B*_ are the respective diffusion coefficients and *a*_0_ is constant. Using the below mentioned transformations

$$\begin{array}{c} \eta =\sqrt{\frac{U_{0}}{\nu l}}\left(\frac{r^{2}-R^{2}}{2R}\right),\;w=\frac{U_{0}z}{l}f^{\prime }\left(\eta \right),\;u=-\sqrt{\frac{\nu U_{0}}{l}}\frac{R}{r}f\left(\eta \right),\\ \theta \left(\eta \right)=\frac{T-T_{\infty }}{T_{\infty }},\;g\left(\eta \right)=\frac{a}{a_{0}},\;h\left(\eta \right)=\frac{b}{a_{0}}, \end{array}$$ (11)

Eq (6) is identically satisfied while Eqs (7) to (10) are reduced to

$$\begin{array}{c} \left(1+2\gamma \eta \right)\left(1+M\right)f^{\prime \prime \prime }+ff^{\prime \prime }-{\left(f^{\prime }\right)}^{2}+2\gamma \left(1+M\right)f^{\prime \prime }\\ -\frac{4}{3}\left(1+2\gamma \eta \right)M\gamma \lambda f^{\prime \prime }-{\left(1+2\gamma \eta \right)}^{2}\lambda Mf^{\prime \prime 2}f^{\prime \prime \prime }-Ha^{2}f^{\prime }=0, \end{array}$$ (12)

$$\begin{array}{cc} & \left(1+2\gamma \eta \right)\theta^{\prime \prime }+2\gamma \theta^{\prime }+\mathrm{Pr}\left(f\theta^{\prime }+\delta \theta \right)=0, \end{array}$$ (13)

$$\begin{array}{cc} & \frac{1}{Sc}\left(\left(1+2\gamma \eta \right)\Phi^{\prime \prime }+\gamma \Phi^{\prime }\right)+f\Phi^{\prime }-K\Phi \varphi^{2}=0, \end{array}$$ (14)

$$\begin{array}{cc} & \frac{\delta^{*}}{Sc}\left(\left(1+2\gamma \eta \right)\varphi^{\prime \prime }+\gamma \varphi^{\prime }\right)+f\varphi^{\prime }+K\Phi \varphi^{2}=0, \end{array}$$ (15)

$$\begin{array}{c} f\left(0\right)=0,\;\;f^{\prime }\left(0\right)=1,\;\;\theta^{\prime }\left(0\right)=-\alpha \left(1+\theta \left(0\right)\right),\;f^{\prime }\left(\infty \right)=0,\;\theta \left(\infty \right)=0, \end{array}$$ (16)

$$\begin{array}{c} \Phi^{\prime }\left(0\right)=K_{s}\Phi \left(0\right),\;\;\;\Phi \left(\infty \right)\to 1,\;\;\delta^{*}\varphi^{\prime }\left(0\right)=-K_{s}\Phi \left(0\right),\;\;\;\varphi \left(\infty \right)\to 0, \end{array}$$ (17)

where *γ* is the curvature parameter, *M* and *λ* the fluid parameters, *Ha*^2^ Hartman number, Pr Prandtl number, *K* gives the strength of measure of homogeneous reaction, *δ* heat absorption (*δ* < 0) or heat generation (*δ* > 0) parameter, *α* conjugate parameter for Newtonian heating, *Sc* the Schmidt number and *K*_*s*_ measures the strength of heterogeneous reaction. The parameters are defined as follows:

$$\begin{array}{ccc} \gamma & = & {\left(\frac{\nu l}{U_{0}R^{2}}\right)}^{\frac{1}{2}},\;\mathrm{Pr}=\frac{\mu c_{p}}{k},\;M=\frac{1}{\mu \beta c},\;\lambda =\frac{U_{0}^{3}z^{2}}{2l^{3}c^{2}\nu },\;\;K=\frac{k_{1}a_{0}^{2}l}{U_{0}},\;\;\delta^{*}=\frac{D_{A}}{D_{B}}\\ \;\;\delta & = & \frac{Ql}{\rho c_{p}U_{0}},\;Sc=\frac{\nu }{D_{A}},\;K_{s}=\frac{k_{s}}{D_{A}}\sqrt{\frac{\nu }{U_{0}}l},\;\;\alpha =h_{s}\sqrt{\frac{\nu }{U_{0}}l},\;\;Ha^{2}=\frac{\sigma_{1}\beta_{0}^{2}l}{\rho U_{0}}. \end{array}$$ (18)

The diffusion coefficients *D*_*A*_ and *D*_*B*_ are assumed same i. e. δ* = 1, here we have [16]

$$\begin{array}{c} \varphi (\eta )+\Phi (\eta )=1, \end{array}$$

thus Eqs (14), (15) and (17) become

$$\begin{array}{c} \left(1+2\gamma \eta \right)\Phi^{\prime \prime }+\gamma \Phi^{\prime }+Scf\Phi^{\prime }-ScK\Phi {\left(1-\Phi \right)}^{2}=0. \end{array}$$ (19)

The corresponding boundary conditions become

$$\begin{array}{c} \Phi^{\prime }\left(0\right)=K_{s}\Phi \left(0\right),\;\Phi \left(\infty \right)\to 1. \end{array}$$ (20)

Skin friction coefficient and local Nusselt number can be defined as follows:

$$\begin{array}{c} C_{f}=\frac{\tau_{w}}{\rho w_{e}^{2}},\;Nu_{z}=\frac{zq_{w}}{k(T-T_{\infty })},\;\;\;\;\; \end{array}$$ (21)

$$\begin{array}{c} \tau_{w}={\left[\left(\mu +\frac{1}{\beta c}\right)\left(\frac{\partial w}{\partial r}\right)-\frac{1}{6\beta c^{3}}{\left(\frac{\partial w}{\partial r}\right)}^{3}\right]}_{r=R},\;q_{w}=-k{\left(\frac{\partial T}{\partial r}\right)}_{r=R}. \end{array}$$ (22)

Dimensionless forms of skin friction coefficient and local Nusselt number are

$$\begin{array}{c} C_{f}{\text{Re}}_{z}^{1/2}=\left(1+M\right)f^{\prime \prime }\left(0\right)-\frac{\lambda }{3}Mf^{\prime \prime^{3}}\left(0\right),\;Nu_{z}{\text{Re}}_{z}^{-1/2}=\alpha \left(1+\frac{1}{\theta \left(0\right)}\right), \end{array}$$ (23)

where $Re_{z}=\frac{w_{e}z}{\nu }$ denotes the local Reynolds number.

## 3 Homotopic solutions

Homotopy analysis method is derived from the fundamental concept of topology. If one function can be continuously deformed into the other then the functions are said to be homotopic functions. If *f*_1_ and *f*_2_ are two continuous functions which maps from a topological space *X* into topological space *Y* then *f*_1_ is homotopic to *f*_2_ if there exists a continuous map *F*

$$\begin{array}{c} F:\;X\times [0,1]\to Y, \end{array}$$ (24)

such that for each x ∈ X

$$\begin{array}{c} F\left(x,\;0\right)=f_{1}\left(x\right),\;F\left(x,\;1\right)=f_{2}\left(x\right), \end{array}$$ (25)

then the map *F* is called homotopic between *f*_1_(*x*) and *f*_2_(*x*).

Liao [21] in 1992 proposed homotopy analysis method to solve the highly nonlinear equations. It is a continuous deformation or change of a function or equation. Moreover, it is independent of small or large physical parameters. It has many advantages when compared to other methods i.e., (i) it is independent of small or large parameters (ii) it confirms the convergence of series solution (iii) it provides great freedom to choose the base function and linear operator. Such flexibility and freedom assist in solving the highly nonlinear problems. The initial guesses and linear operators for the dimensionless momentum, energy and homogeneous-heterogeneous reactions equations are (*f*_0_, *θ*_0_, Φ_0_) and $\left(L_{f},L_{\theta },L_{\Phi }\right)$ The initial guesses and linear operators are taken as follows:

$$\begin{array}{c} f_{0}\left(\eta \right)=1-\exp\left(-\eta \right),\;\;\;\;\theta_{0}\left(\eta \right)=\frac{\alpha }{1-\alpha }\exp\left(-\eta \right),\;\Phi_{0}\left(\eta \right)=1-\frac{1}{2}\exp\left(-k_{s}\eta \right), \end{array}$$ (26)

$$\begin{array}{c} L_{f}\left(\eta \right)=\frac{d^{3}f}{d\eta^{3}}-\frac{df}{d\eta },\;\;\;\;L_{\theta }\left(\eta \right)=\frac{d^{2}\theta }{d\eta^{2}}-\theta ,\;L_{\Phi }\left(\eta \right)=\frac{d^{2}\Phi }{d\eta^{2}}-\Phi , \end{array}$$ (27)

$$\begin{array}{c} L_{f}\left[A_{1}+A_{2}\exp\left(\eta \right)+A_{3}\exp\left(-\eta \right)\right]=0, \end{array}$$ (28)

$$\begin{array}{c} L_{\theta }\left[A_{4}\exp\left(\eta \right)+A_{5}\exp\left(-\eta \right)\right]=0, \end{array}$$ (29)

$$\begin{array}{c} L_{\Phi }\left[A_{6}\exp\left(\eta \right)+A_{7}\exp\left(-\eta \right)\right]=0, \end{array}$$ (30)

where *A*_*i*_ (*i* = 1 − 7) are the arbitrary constants.

### 3.1 Zeroth-order problems

$$\begin{array}{c} \left(1-p\right)L_{f}\left[\hat{f}\left(\eta ;p\right)-f_{0}\left(\eta \right)\right]=p\hbar_{f}N_{f}\left[\hat{f}\left(\eta ;p\right)\right], \end{array}$$ (31)

$$\begin{array}{c} \left(1-p\right)L_{\theta }\left[\hat{\theta }\left(\eta ;p\right)-\theta_{0}\left(\eta \right)\right]=p\hbar_{\theta }N_{\theta }\left[\hat{\theta }\left(\eta ;p\right),\hat{f}\left(\eta ;p\right)\right], \end{array}$$ (32)

$$\begin{array}{c} \left(1-p\right)L_{\Phi }\left[\hat{\Phi }\left(\eta ;p\right)-\Phi_{0}\left(\eta \right)\right]=p\hbar_{\Phi }N_{\Phi }\left[\hat{\Phi }\left(\eta ;p\right),\hat{f}\left(\eta ;p\right)\right], \end{array}$$ (33)

$$\begin{array}{c} \hat{f}\left(0;p\right)=0,\;\;{\hat{f}}^{\prime }\left(0;p\right)=1,\;{\hat{\theta }}^{\prime }\left(0;p\right)=-\alpha \left(1+\hat{\theta }\left(0;p\right)\right),\;\;{\hat{\Phi }}^{\prime }\left(0;p\right)=K_{s}\hat{\Phi }\left(0;p\right), \end{array}$$ (34)

$$\begin{array}{c} \;{\hat{f}}^{\prime }\left(\eta ;p\right)\to 0\;\;\;\;\hat{\theta }\left(\eta ;p\right)\to 0,\;\;\;\hat{\Phi }\left(\eta ;p\right)\to 1\;\text{as}\;\;\eta \to \infty , \end{array}$$ (35)

$$\begin{array}{ll} N_{f}\left[\hat{f}\left(\eta ,p\right),\hat{\theta }\left(\eta ;p\right)\right] & =\left(1+2\gamma \eta \right)(1+M)\frac{\partial^{3}\hat{f}\left(\eta ;p\right)}{\partial \eta^{3}}+2\gamma (1+M)\frac{\partial^{2}\hat{f}\left(\eta ;p\right)}{\partial \eta^{2}}\\ & +\frac{\partial^{2}\hat{f}\left(\eta ;p\right)}{\partial \eta^{2}}\hat{f}\left(\eta ;p\right)-{\left(1+2\gamma \eta \right)}^{2}M\lambda {\left(\frac{\partial^{2}\hat{f}\left(\eta ;p\right)}{\partial \eta^{2}}\right)}^{2}\frac{\partial^{3}\hat{f}\left(\eta ;p\right)}{\partial \eta^{3}}n\\ & -{\left(\frac{\partial \hat{f}\left(\eta ;p\right)}{\partial \eta }\right)}^{2}+\frac{4}{3}\left(1+2\gamma \eta \right)M\gamma \lambda {\left(\frac{\partial^{2}\hat{f}\left(\eta ;p\right)}{\partial \eta^{2}}\right)}^{3}-Ha^{2}\frac{\partial \hat{f}\left(\eta ;p\right)}{\partial \eta }, \end{array}$$ (36)

$$\begin{array}{ccc} N_{\theta }\left[\hat{\theta }\left(\eta ;p\right),\hat{f}\left(\eta ;p\right)\right] & = & \left(1+2\gamma \eta \right)\frac{\partial^{2}\hat{\theta }\left(\eta ,p\right)}{\partial \eta^{2}}+2\gamma \frac{\partial \hat{\theta }\left(\eta ,p\right)}{\partial \eta }\\ & & +\mathrm{Pr}\left(\hat{f}\left(\eta ;p\right)\frac{\partial \hat{\theta }\left(\eta ;p\right)}{\partial \eta }\right)+\mathrm{Pr}\delta \hat{\theta }\left(\eta ;p\right), \end{array}$$ (37)

$$\begin{array}{ccc} N_{\Phi }\left[\hat{\Phi }\left(\eta ;p\right),\hat{f}\left(\eta ;p\right)\right] & = & \left(1+2\gamma \eta \right)\frac{\partial^{2}\hat{\Phi }\left(\eta ,p\right)}{\partial \eta^{2}}+2\gamma \frac{\partial \hat{\Phi }\left(\eta ,p\right)}{\partial \eta }\\ & & +Sc\left(\hat{f}\left(\eta ;p\right)\frac{\partial \hat{\Phi }\left(\eta ;p\right)}{\partial \eta }\right)-KSc\left(\hat{\Phi }\left(\eta ;p\right)\right)\\ & & +2KSc{\left(\hat{\Phi }\left(\eta ;p\right)\right)}^{2}-KSc{\left(\hat{\Phi }\left(\eta ;p\right)\right)}^{3}, \end{array}$$ (38)

where *p* ∈ [0, 1] is embedding parameter and ℏ_*f*_, ℏ_*θ*_ and ℏ_Φ_ are the non-zero auxiliary parameters.

### 3.2 m-order deformation problems

$$\begin{array}{c} L_{f}\left[f_{m}\left(\eta \right)-\chi_{m}f_{m-1}\left(\eta \right)\right]=\hbar_{f}R_{m}^{f}\left(\eta \right), \end{array}$$ (39)

$$\begin{array}{c} L_{\theta }\left[\theta_{m}\left(\eta \right)-\chi_{m}\theta_{m-1}\left(\eta \right)\right]=\hbar_{\theta }R_{m}^{\theta }\left(\eta \right), \end{array}$$ (40)

$$\begin{array}{c} L_{\Phi }\left[\Phi_{m}\left(\eta \right)-\chi_{m}\Phi_{m-1}\left(\eta \right)\right]=\hbar_{\Phi }R_{m}^{\Phi }\left(\eta \right), \end{array}$$ (41)

$$\begin{array}{c} f_{m}^{\prime }\left(0\right)=0,\;\text{and}\;f_{m}^{\prime }\left(\infty \right)\to 0\;\text{as}\;\eta \to \infty , \end{array}$$ (42)

$$\begin{array}{c} \alpha \theta_{m}\left(0\right)+\theta_{m}^{\prime }\left(0\right)=0,\;\text{and}\;\theta_{m}\left(\infty \right)\to 0\;\text{as}\;\eta \to \infty , \end{array}$$ (43)

$$\begin{array}{c} \Phi_{m}^{\prime }\left(0\right)=K_{s}\phi_{m}\left(0\right),\;\text{and}\;\Phi_{m}\left(\infty \right)\to 0\;\text{as}\;\eta \to \infty . \end{array}$$ (44)

$$\begin{array}{ll} R_{m}^{f}\left(\eta \right) & =\left(1+2\gamma \eta \right)\left(1+M\right)f_{m-1}^{\prime \prime \prime }+2\gamma \left(1+M\right)f_{m-1}^{\prime \prime }-Ha^{2}\Phi f_{m-1}^{\prime }+\sum_{k=0}^{m-1}\left[f_{m-1-k}f_{k}^{\prime \prime }-f_{m-1-k}^{\prime }f_{k}^{\prime }\right]\\ & +\frac{4}{3}\left(1+2\gamma \eta \right)M\lambda \gamma \sum_{k=0}^{m-1}f_{m-1-k}^{{}^{\prime \prime \prime }}\sum_{l=0}^{k}f_{k-l}^{\prime \prime \prime }f_{l}^{\prime \prime \prime }-{\left(1+2\gamma \eta \right)}^{2}M\lambda \sum_{k=0}^{m-1}f_{m-1-k}^{\prime \prime }\sum_{l=0}^{k}f_{k-l}^{\prime \prime }f_{l}^{\prime \prime \prime }, \end{array}$$ (45)

$$\begin{array}{cc} R_{m}^{\theta }\left(\eta \right) & =\left(1+2\gamma \eta \right)\theta_{m-1}^{\prime \prime }+2\gamma \theta_{m-1}^{\prime }+\sum_{k=0}^{m-1}\mathrm{Pr}f_{m-1-k}\theta_{k}^{\prime }+\mathrm{Pr}\delta \theta_{m-1} \end{array}$$ (46)

$$\begin{array}{ll} R_{m}^{\Phi }\left(\eta \right) & =\left(1+2\gamma \eta \right)\Phi_{m-1}^{\prime \prime }+2\gamma \Phi_{m-1}^{\prime }+\sum_{k=0}^{m-1}Scf_{m-1-k}^{\prime }\Phi_{k}\\ & -\sum_{k=0}^{m-1}(\Phi_{m-1-k}(ScK-2ScK\Phi_{k}+ScK\sum_{l=0}^{k}\Phi_{k-l}\Phi_{k}), \end{array}$$ (47)

$$\begin{array}{c} \chi_{m}=\left\{\begin{array}{c} 0,\;\;\;m\leq 1\\ 1,\;\;\;m>1 \end{array}.\right. \end{array}$$ (48)

Employing homotopic procedure [21] the solutions are

$$\begin{array}{c} f_{m}\left(\eta \right)=f_{m}^{⋆}\left(\eta \right)+A_{1}+A_{2}e^{\eta }+A_{3}e^{-\eta }, \end{array}$$ (49)

$$\begin{array}{c} \theta_{m}\left(\eta \right)=\theta_{m}^{⋆}\left(\eta \right)+A_{4}e^{\eta }+A_{5}e^{-\eta }, \end{array}$$ (50)

$$\begin{array}{c} \Phi_{m}\left(\eta \right)=\Phi_{m}^{⋆}\left(\eta \right)+A_{6}e^{\eta }+A_{7}e^{-\eta }, \end{array}$$ (51)

where the constants *A*_*i*_ (*i* = 1–7) have the values:

$$\begin{array}{ccc} A_{2} & = & A_{4}=A_{6}=0,\;\;\;\;\;\;A_{3}={\left.\frac{\partial f_{m}^{⋆}\left(\eta \right)}{\partial \eta }\right|}_{\eta =0}\text{,}\;\;\;\;\;\;A_{1}=-A_{3}-f_{m}^{⋆}\left(0\right)\text{,}\;\;\;\;\;\;\;\\ A_{5} & = & \frac{1}{1-\alpha }\left(\alpha \left(\theta_{m}^{⋆}\left(0\right)\right)+\theta_{m}^{{}^{\prime }⋆}\left(0\right)\right),\;A_{7}=\frac{1}{1+K_{s}}\left(\Phi_{m}^{\prime ⋆}\left(0\right)-K_{s}\Phi_{m}^{⋆}\left(0\right)\right). \end{array}$$ (52)

**Fig 2:**
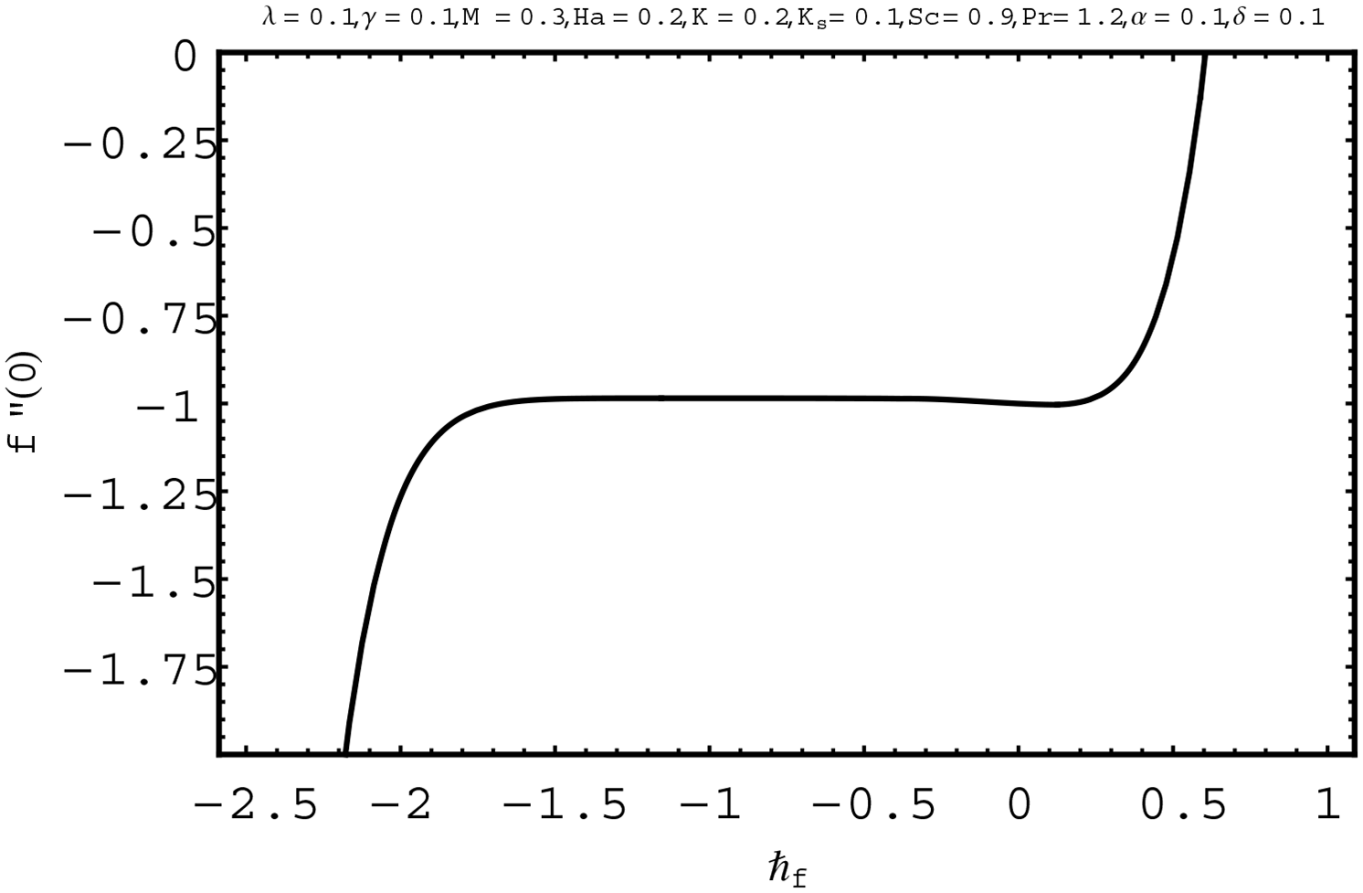
ℏ− curve for *f*(*η*).

**Fig 3:**
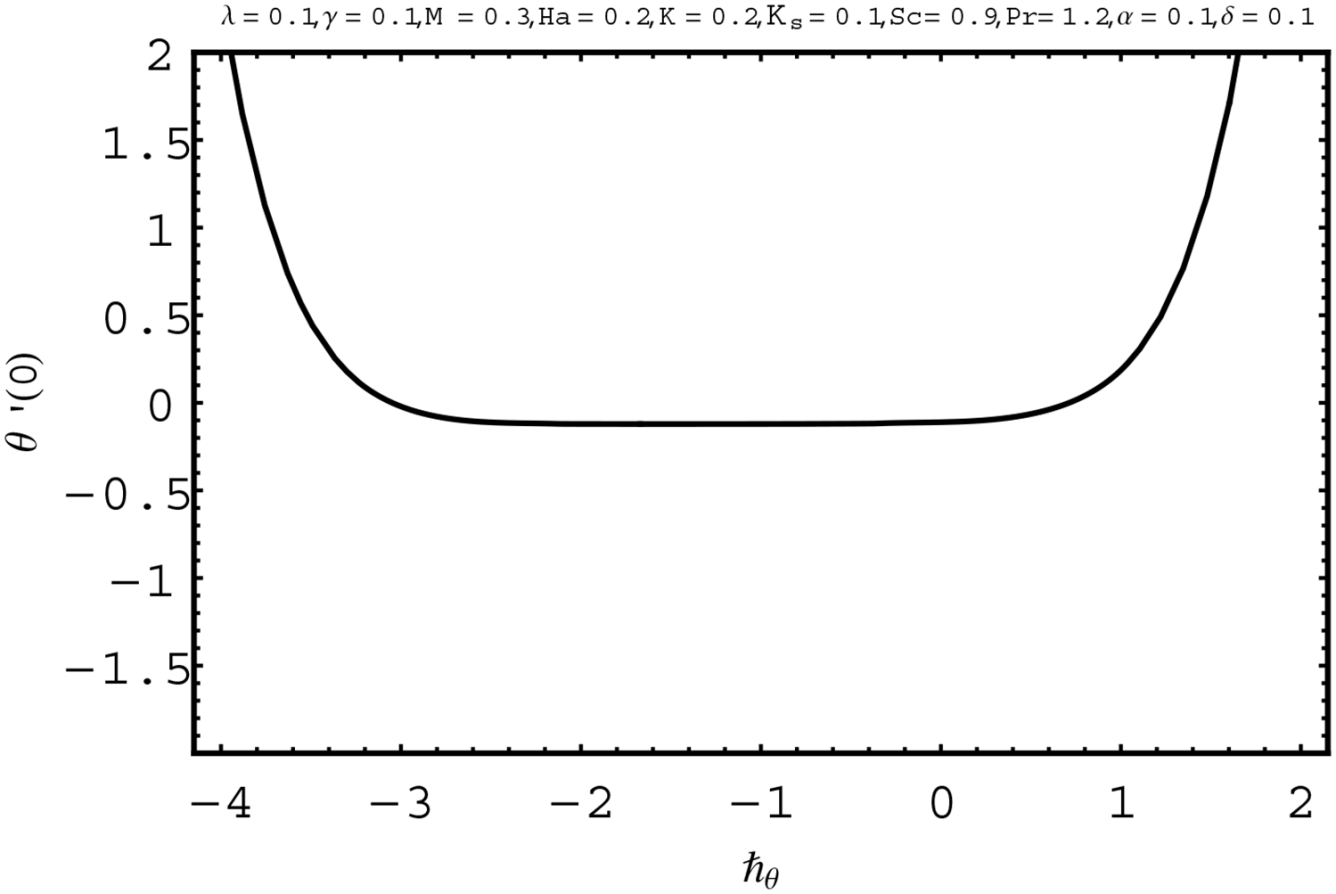
ℏ− curve for *θ*(*η*).

**Fig 4:**
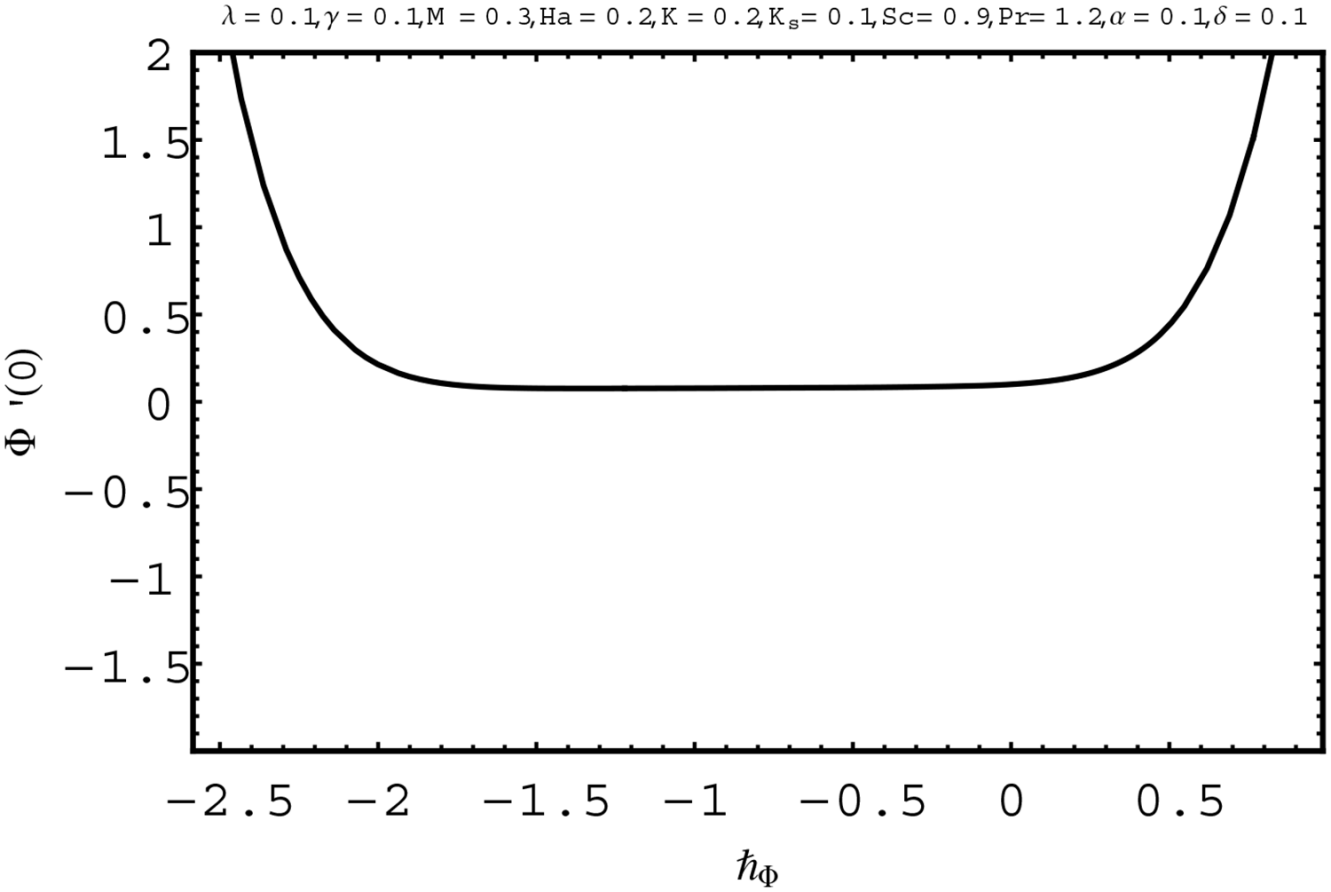
ℏ− curve for Φ(*η*).

For the series solutions of momentum, energy and homogeneous-heterogeneous reactions equations by homotopy analysis method, the convergence region is essential. Convergence region of the series solutions depend upon the auxiliary parameter ℏ. Therefore we have plotted the ℏ-curves in Figs 2 to 4. The admissible ranges of the auxiliary parameters ℏ_*f*_, ℏ_*θ*_ and ℏ_Φ_ are −1.5 ≤ ℏ_*f*_ ≤ −0.1, −2.5 ≤ ℏ_*θ*_ ≤ −0.1 and −1.9 ≤ ℏ_Φ_ ≤ −0.8 respectively.

## 4 Discussion

**Fig 5:**
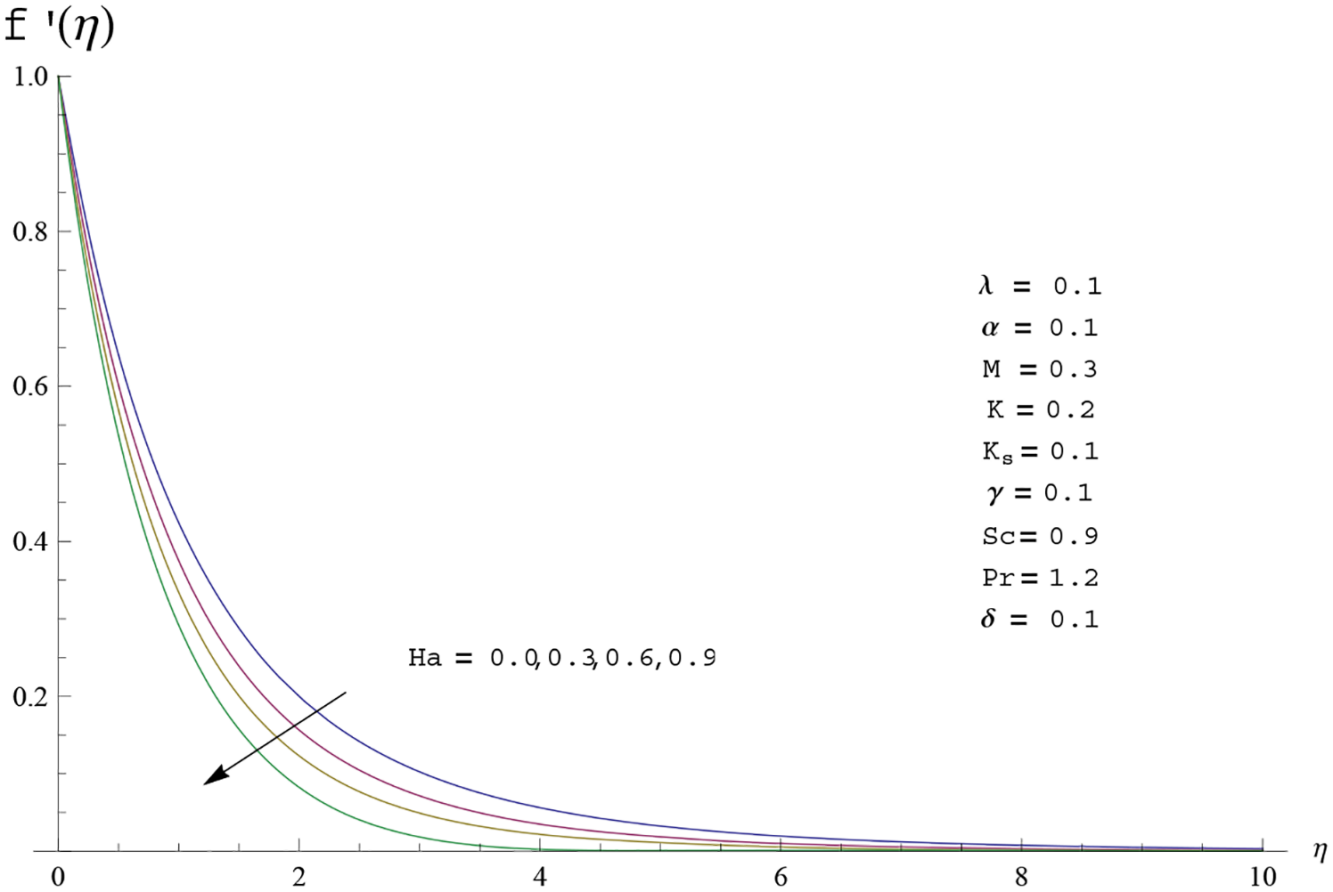
Velocity profile *f*′(*η*) for different values of Hartman number Ha.

**Fig 6:**
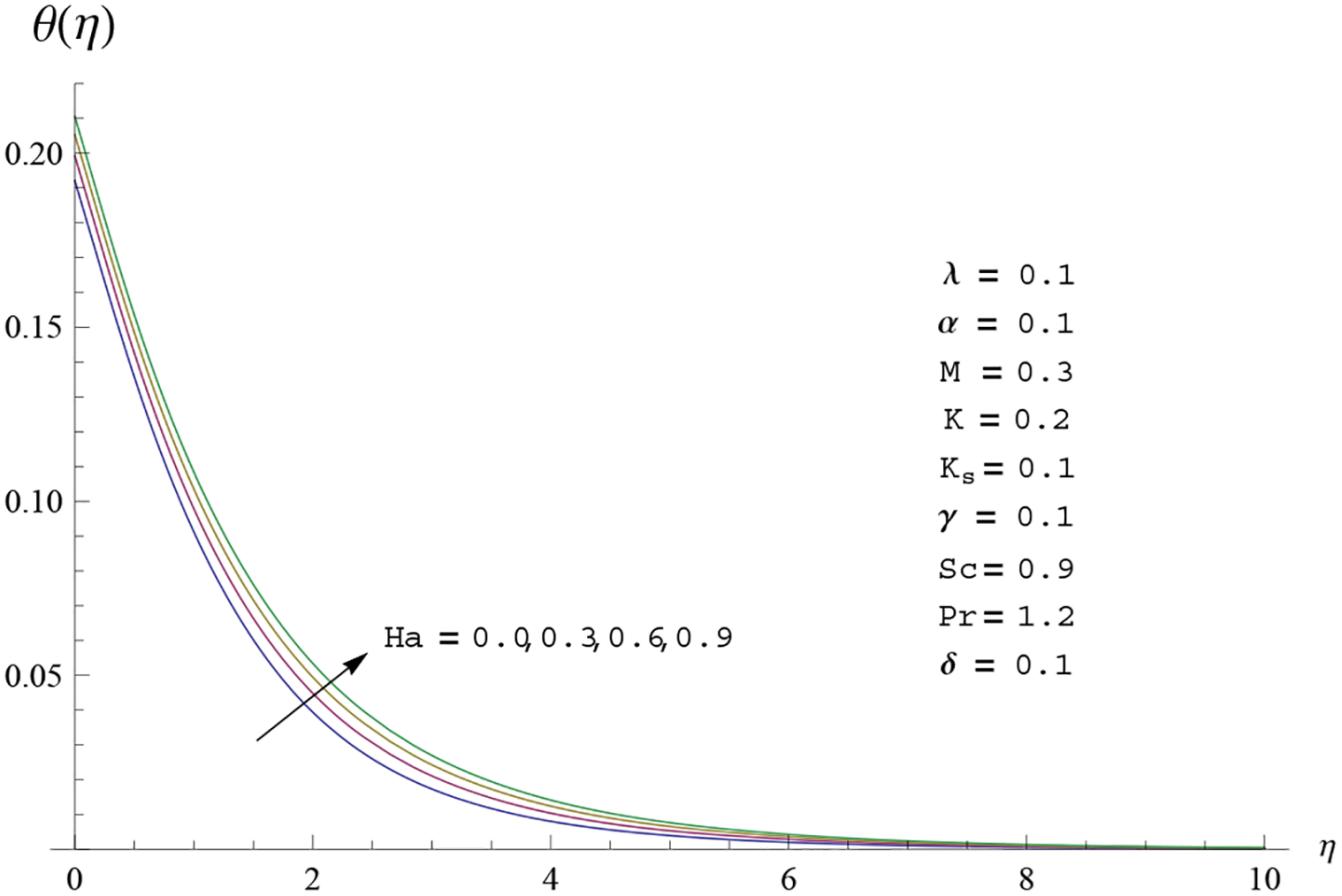
Temperature profile *θ*(*η*) for different values of Hartman number Ha.

**Fig 7:**
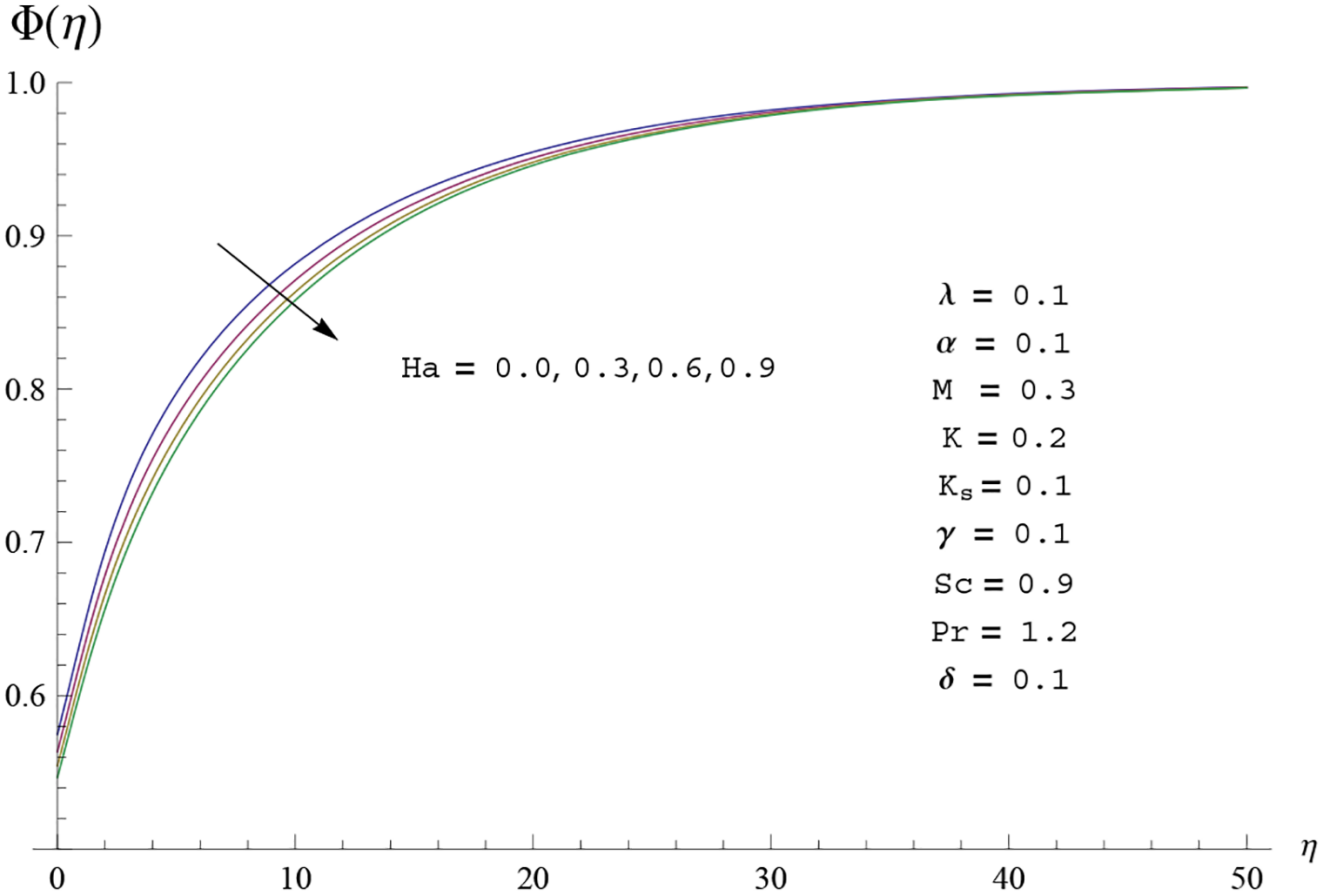
Concentration profile Φ(*η*) for different values Hartman number Ha.

**Fig 8:**
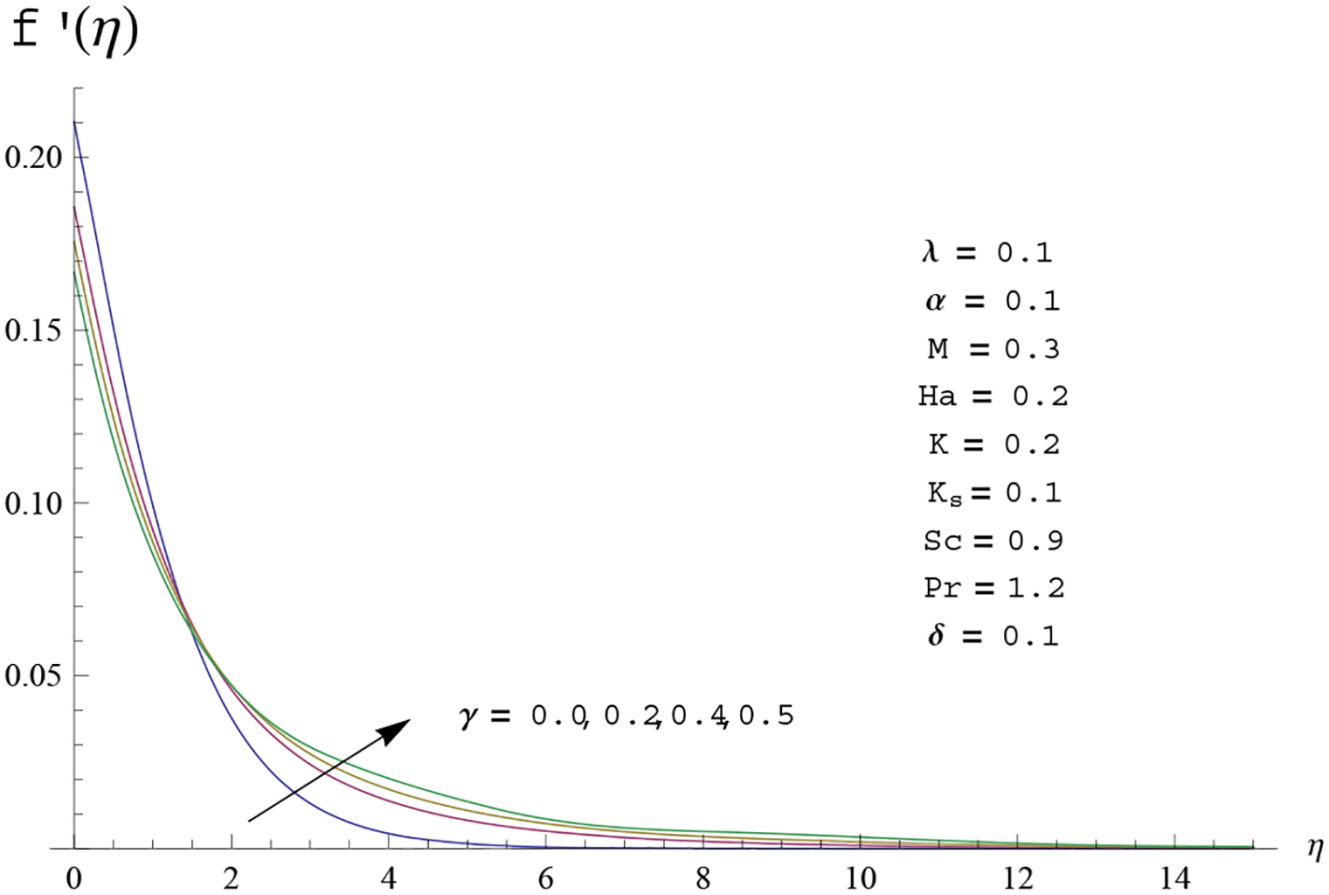
Velocity profile *f*′(*η*) for different values of curvature parameter *γ*.

**Fig 9:**
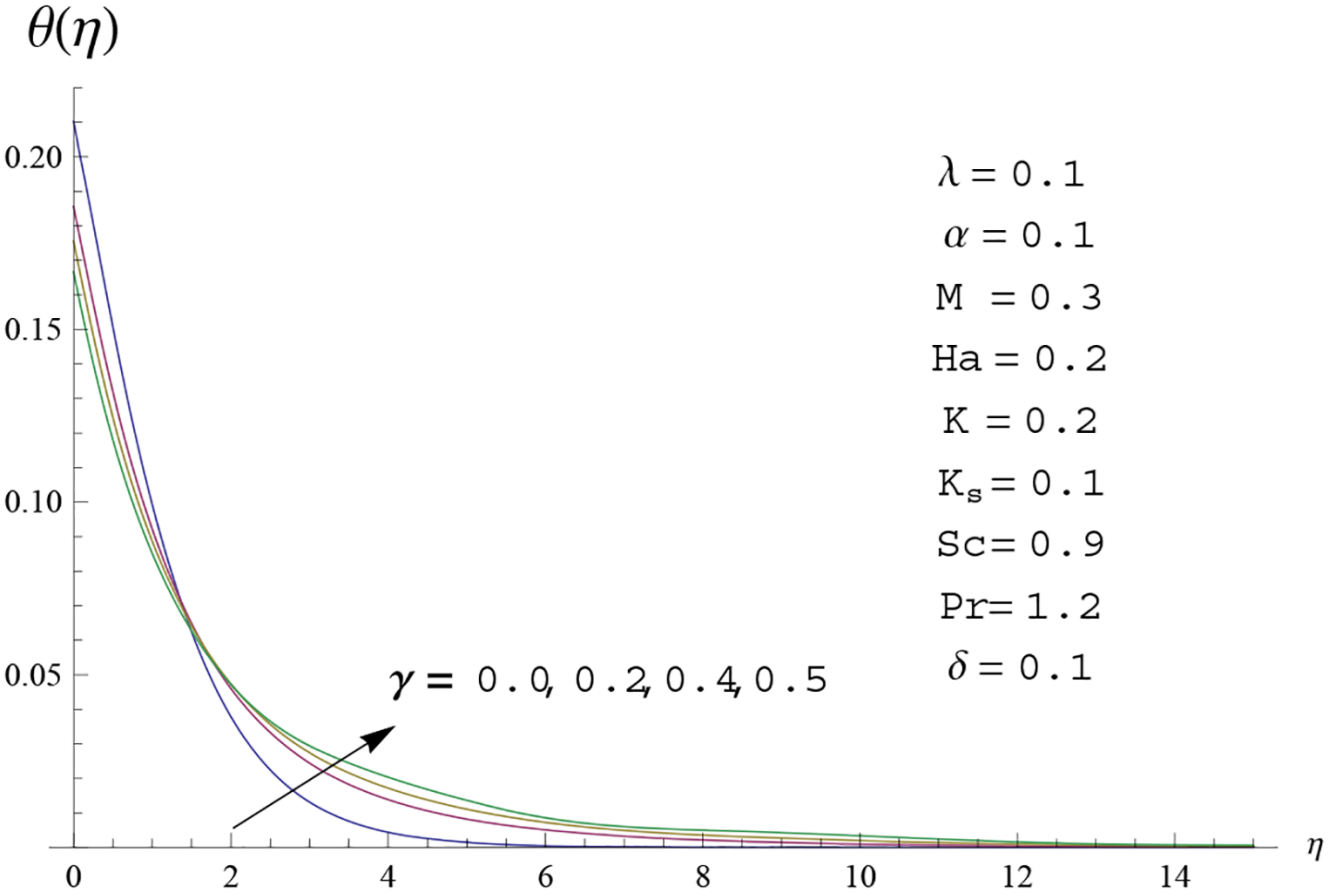
Temperature profile *θ*(*η*) for different values of curvature parameter *γ*.

**Fig 10:**
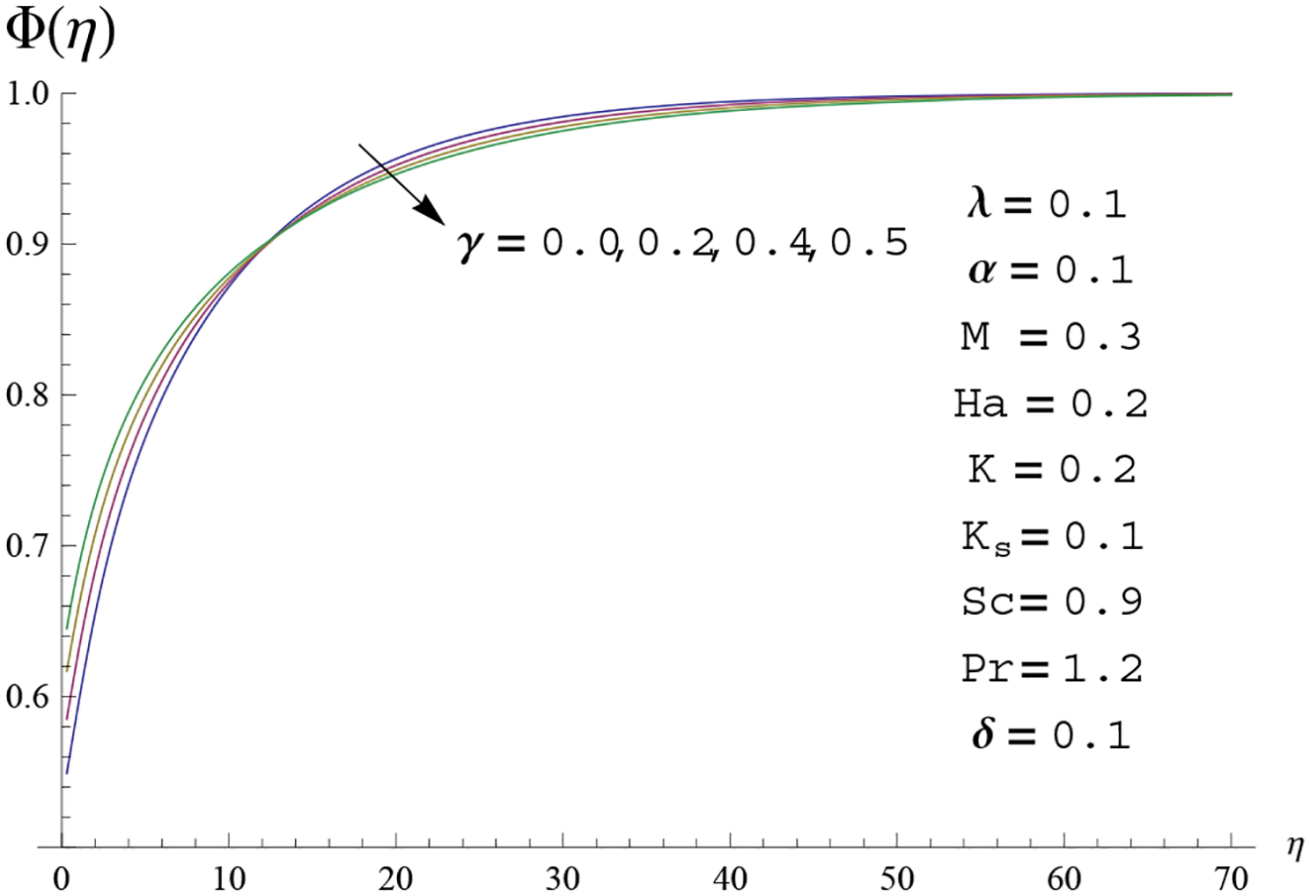
Concentration profile Φ(*η*) for different values of curvature parameter *γ*.

**Fig 11:**
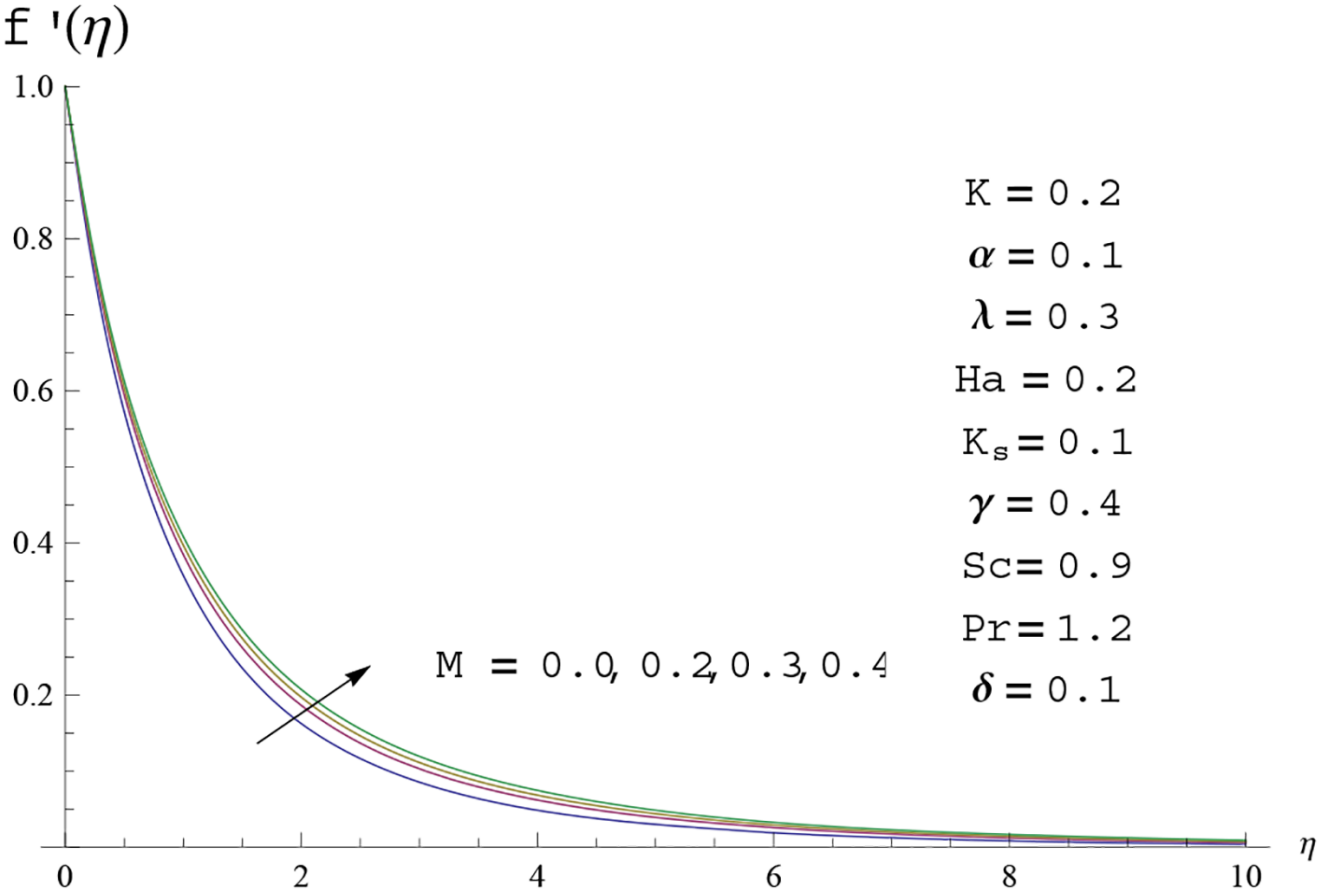
Velocity profile *f*′(*η*) for different values of fluid parameter M.

**Fig 12:**
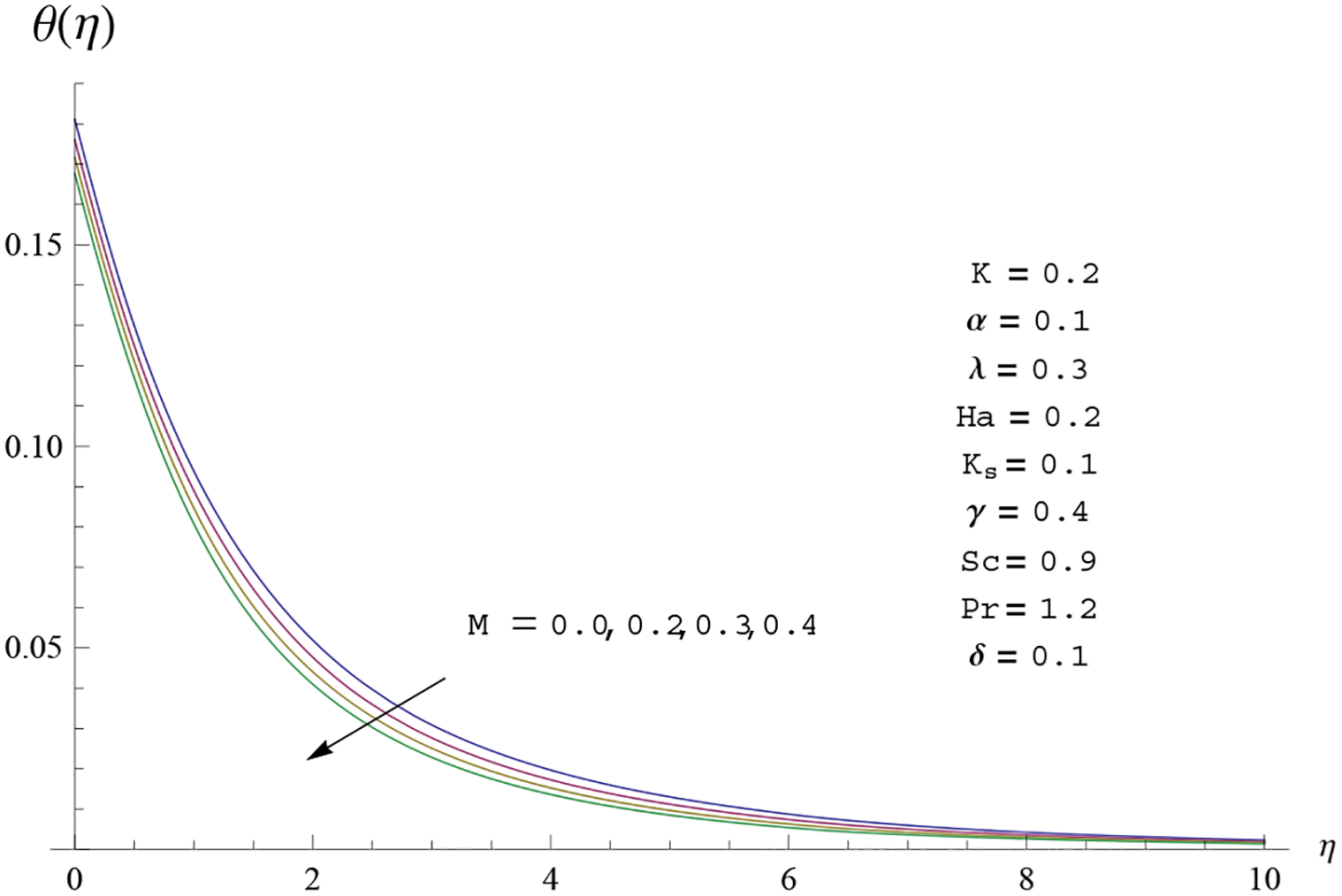
Temperature profile *θ*(*η*) for different values of fluid parameter M.

**Fig 13:**
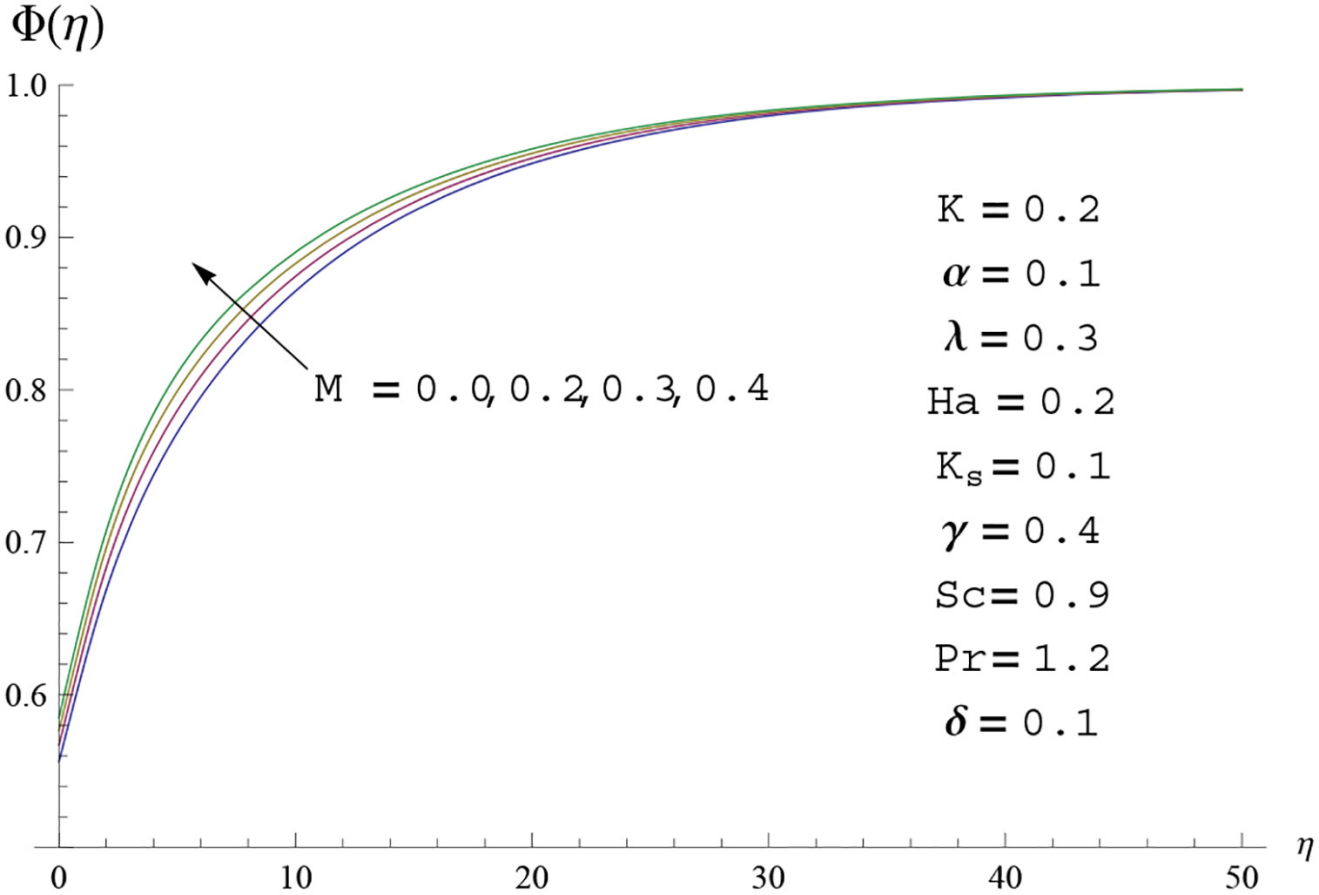
Concentration profile Φ(*η*) for different values of fluid parameter M.

**Fig 14:**
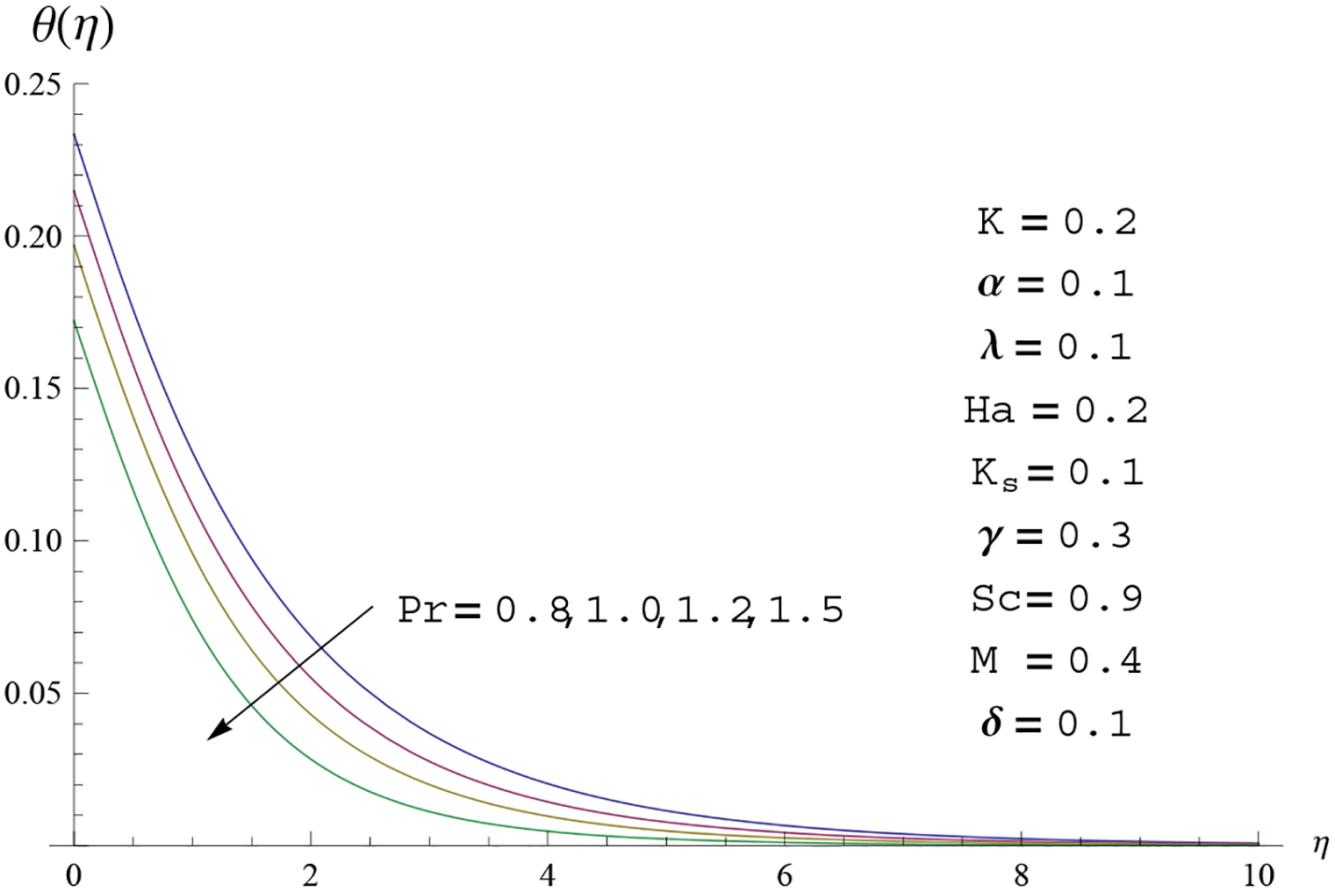
Temperature profile *θ*(*η*) for different values of Prandtl number *Pr*.

**Fig 15:**
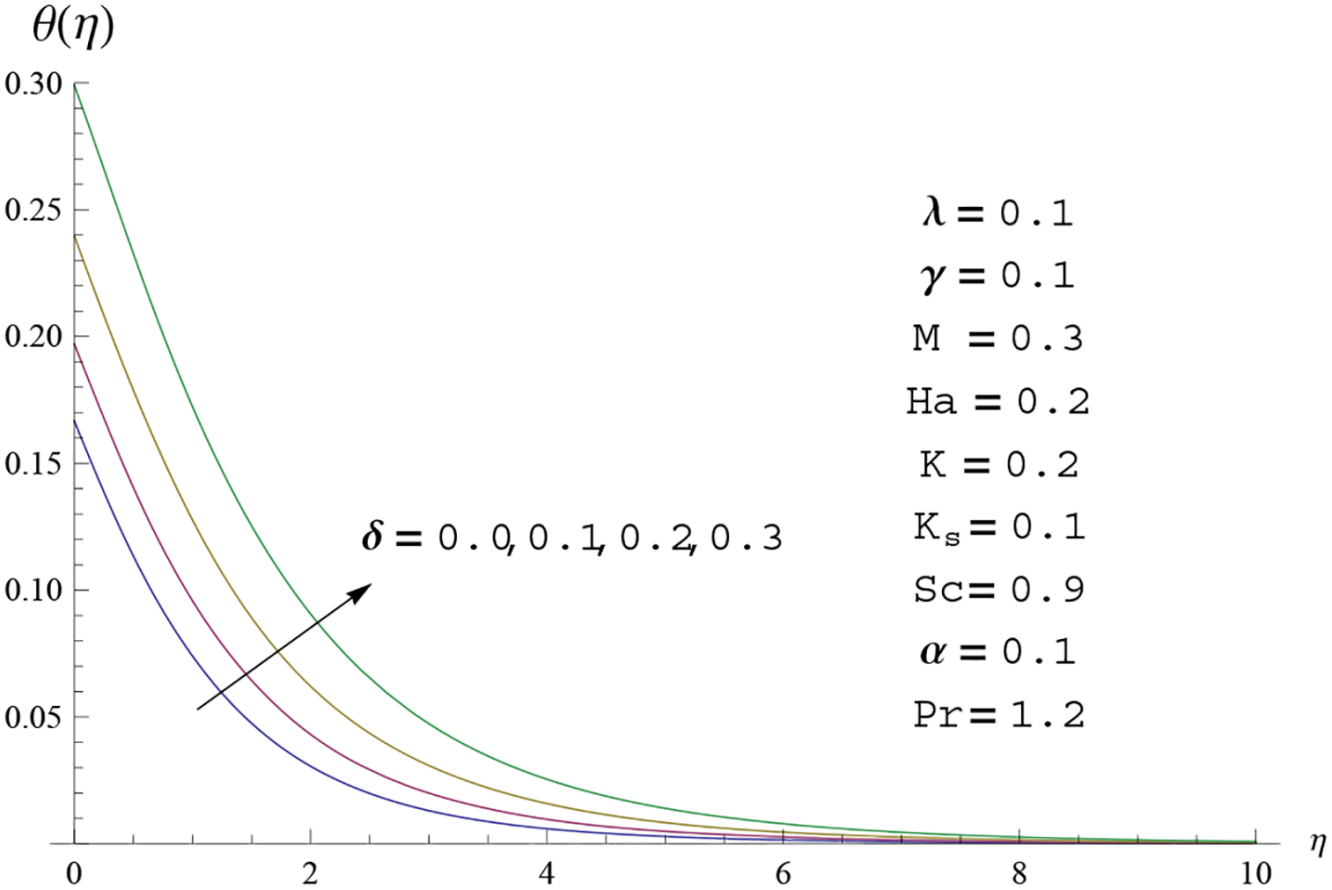
Temperature profile *θ*(*η*) for different values of heat generation parameter *δ*.

**Fig 16:**
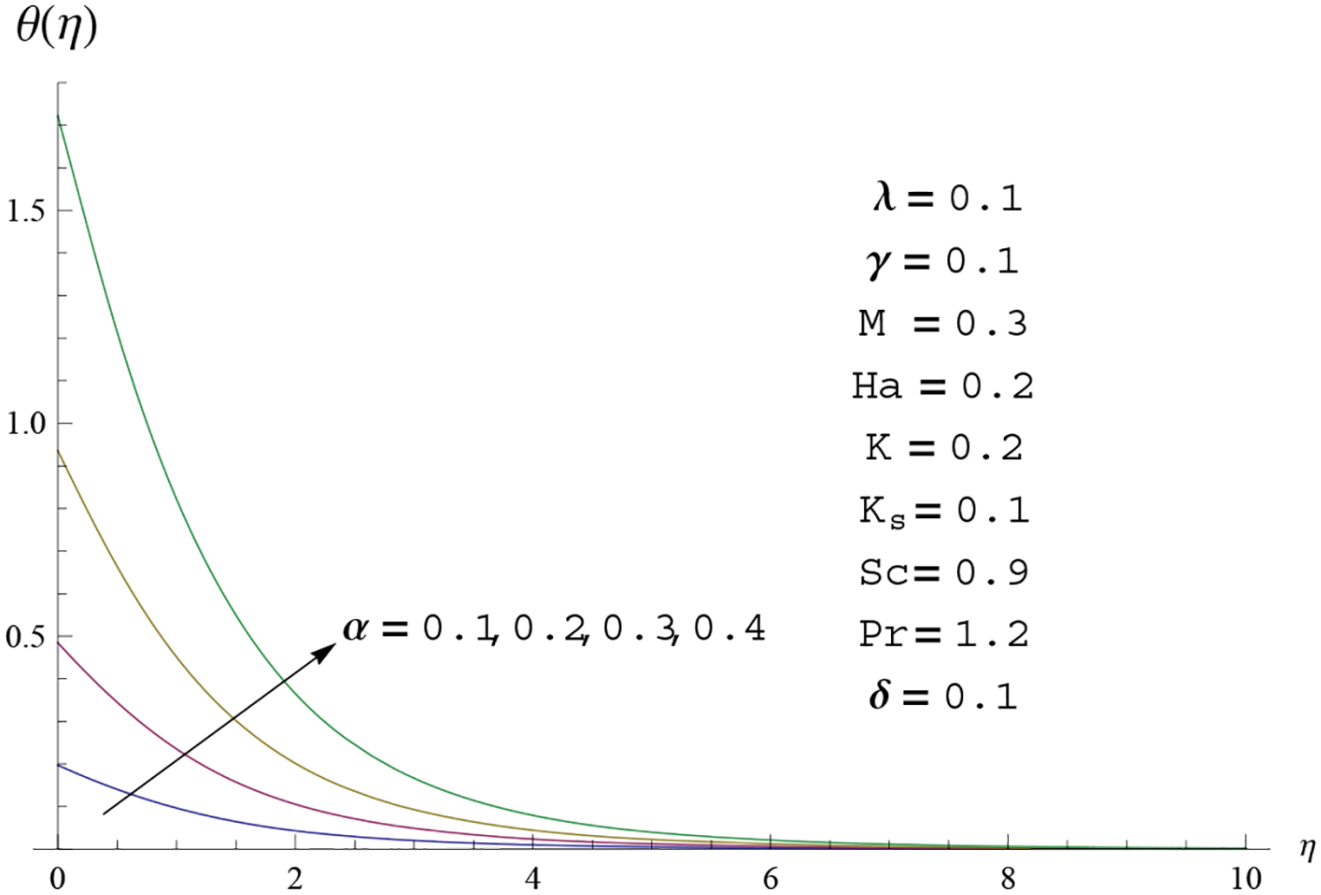
Temperature profile *θ*(*η*) for different values of conjugate parameter *α*.

**Fig 17:**
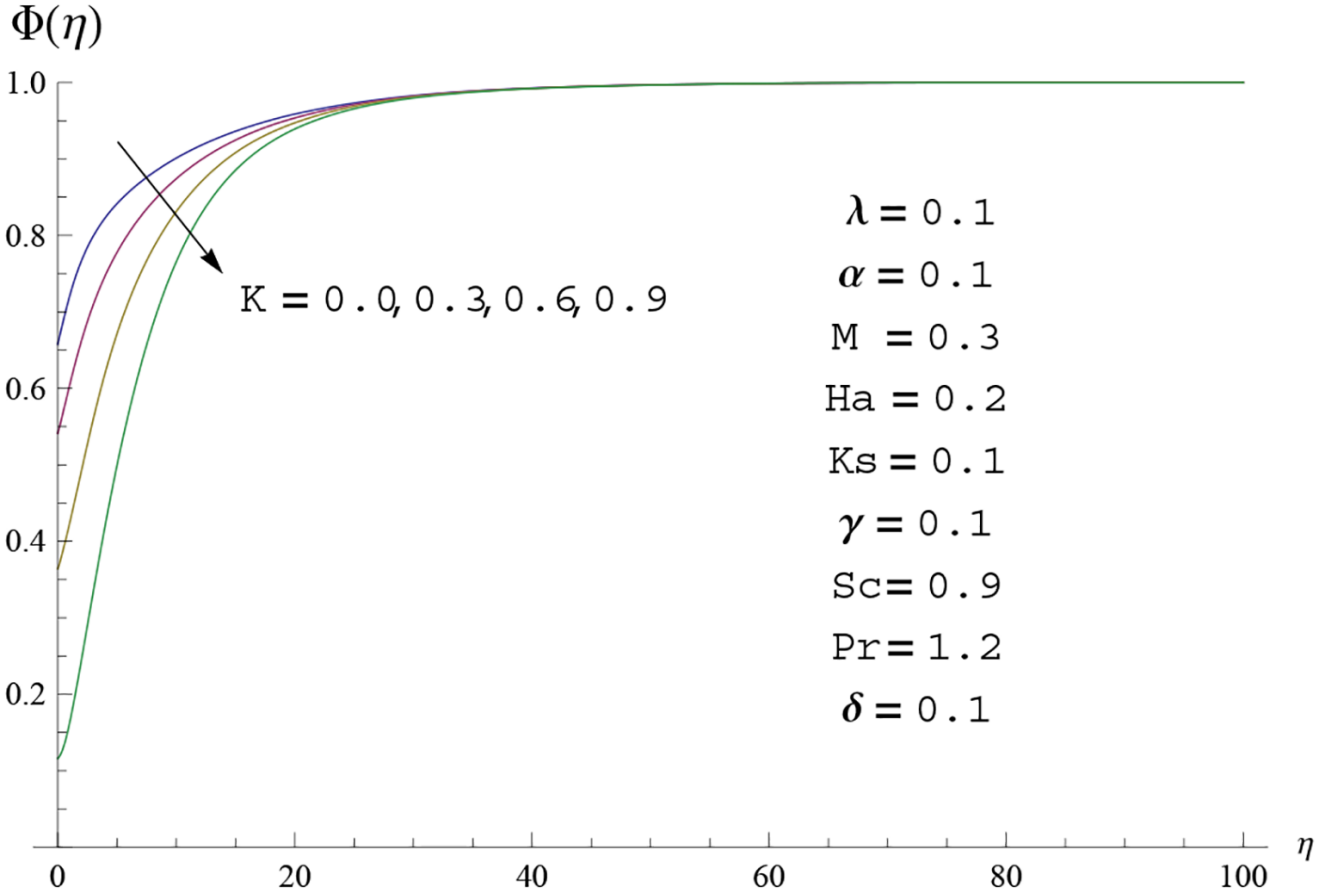
Concentration profile Φ(*η*) for different values of homogeneous parameter *K*.

**Fig 18:**
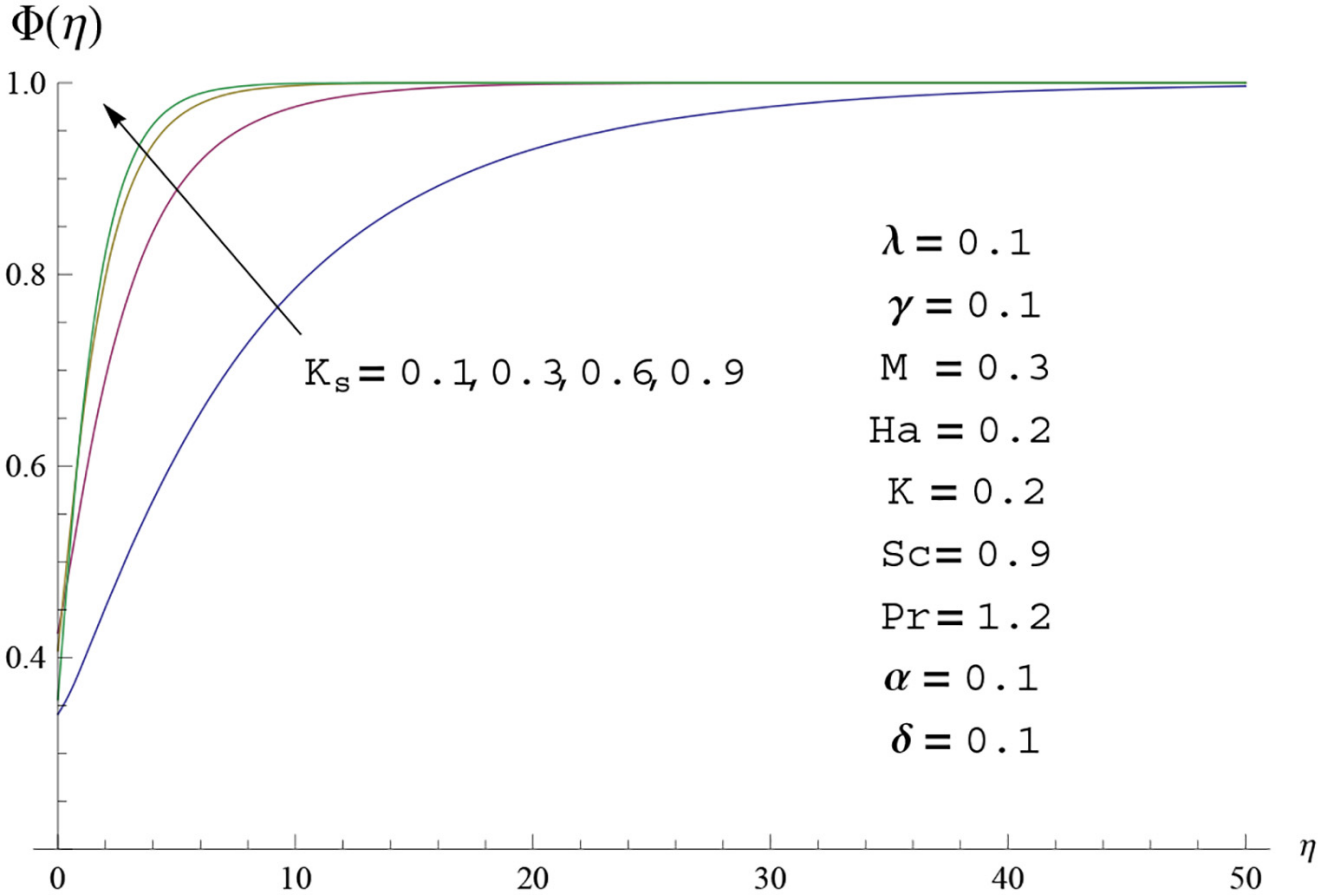
Concentration profile Φ(*η*) for different values of heterogeneous parameter *K*_*s*_.

**Fig 19:**
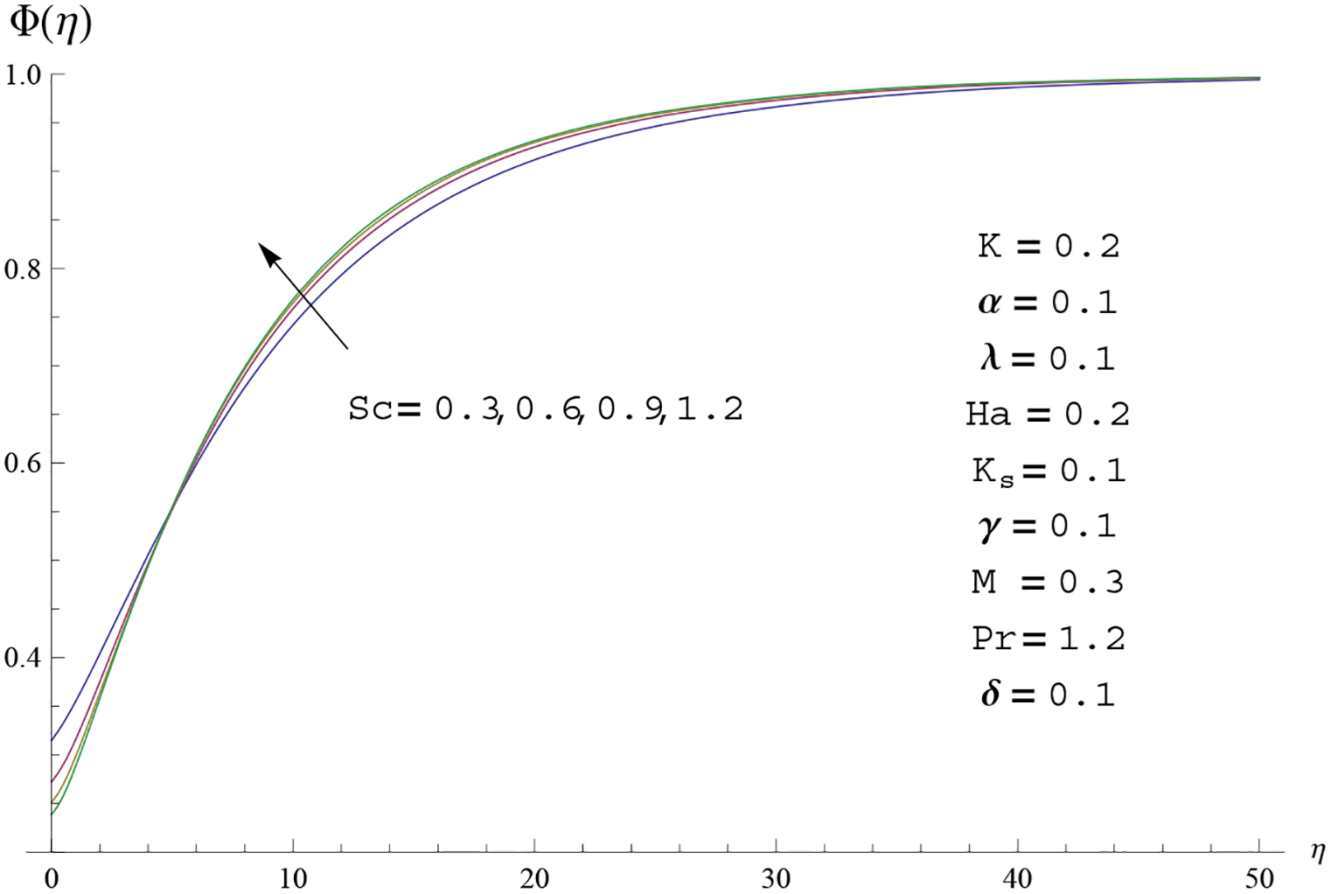
Concentration profile Φ(*η*) for different values of Schmidt number *Sc*.

Interest in present section is to examine the effects of different parameters on the velocity, temperature and concentration profiles. Fig 5 is plotted for the effect of Hartman number *Ha*^2^ on the velocity profile *f*′(*η*). Here both velocity and layer thickness decrease for larger values of Hartman number *Ha*^2^. In fact an increase in values of Hartman number *Ha*^2^ shows stronger Lorentz force which offers more resistance to the fluid motion and thus the velocity profile *f*′(*η*) decreases. Fig 6 is sketched for the influence of *Ha*^2^ on temperature profile *θ*(*η*). It is observed that both temperature and thermal boundary layer thickness increase for larger values of Hartman number *Ha*^2^. Fig 7 is plotted for concentration profile Φ(*η*). The concentration profile Φ(*η*) is found to decrease when Hartman number *Ha*^2^ increases. Fig 8 is sketched for the effect of curvature parameter *γ* on the velocity profile *f*′(*η*). It is noted that for larger curvature parameter *γ* the velocity profile and momentum boundary layer thickness first decrease and then increase. This is due to the fact that for increase in curvature parameter *γ* the radius of cylinder decreases and consequently less resistive force occurs for the fluid and the velocity profile *f*′(*η*) increases. Similar behavior is observed for temperature profile *θ*(*η*) (see Fig 9). It is analyzed that temperature profile *θ*(*η*) decreases near the surface of cylinder while it increases away from the surface. For higher values of curvature parameter *γ*, the radius of cylinder decreases and consequently less particles are sticky to the surface which are responsible for heat transfer through conduction. Therefore temperature profile decreases near the surface of cylinder. It is also observed that temperature profile is higher in case of flat plate when compared with the cylinder. Fig 10 is sketched for the effect of curvature parameter *γ* on concentration profile Φ(*η*). It is noted here that through larger curvature parameter *γ*, the thermal boundary layer thickness first decreases and then increases. For curvature parameter *γ* = 0 the problem reduces to flat plate. Fig 11 is sketched for different values of fluid parameter *M*. Both velocity and associated boundary layer increase via *M*. As *M* increases the fluid becomes less viscous which enhances the fluid velocity. However the temperature and thermal boundary layer thickness decrease via *M* (see Fig 12). This is due to fact that as *M* increases, fluid becomes less viscous. Therefore the thermal boundary layer thickness and temperature profile decrease. Fig 13 is sketched for the behavior of fluid parameter *M* on concentration profile Φ(*η*). Clearly the concentration profile Φ(*η*) is an increasing function of *M*. Effect of Prandtl number Pr on temperature profile *θ*(*η*) is displayed in Fig 14. It is analyzed that both temperature and thermal layer thickness are decreasing functions of Pr. It is observed that due to increase in Prandtl number Pr the thermal diffusivity decreases. As a result the temperature and thermal boundary layer thickness decrease. Fig 15 is sketched for the effect of heat generation parameter *δ* on temperature profile *θ*(*η*). It is observed that for increase in heat generation parameter *δ* the thermal boundary layer and temperature of the fluid are enhanced. In fact more heat is produced due to increase in heat generation parameter. Hence temperature profile *θ*(*η*) increases. Fig 16 is plotted for the variation of conjugate parameter *α* on temperature profile *θ*(*η*). Here temperature and thermal boundary layer thickness are increasing functions of conjugate parameter *α*. Higher values of conjugate parameter results in higher heat transfer coefficient which consequently enhances the fluid temperature and also the thermal boundary layer thickness. Effect of strength of homogeneous reaction *K* on concentration distribution Φ(*η*) is plotted in Fig 17. Concentration profile Φ(*η*) decreases while boundary layer thickness increases for higher values of strength of homogeneous reaction parameter. Behavior of strength of heterogeneous reaction parameter *K*_*s*_ on concentration distribution Φ(*η*) is computed in Fig 18. Concentration distribution Φ(*η*) increases for higher values of heterogeneous parameter *K*_*s*_. Influence of Schmidt number *Sc* on concentration profile Φ(*η*) is shown in Fig 19. Increasing behavior of concentration profile Φ(*η*) is observed for higher values of Schmidt number *Sc* away from the surface of cylinder. It is due to the fact that Schmidt number *Sc* is the ratio of momentum diffusivity to mass diffusivity as a result higher values of Schmidt number *Sc* correspond to small mass diffusivity. Hence concentration profile Φ(*η*) decreases. Table 1 shows the convergence of the series solutions for dimensionless momentum, energy and concentration equations. It is observed that 25^th^ orders of approximations are sufficient for the convergence of required equations. Table 2 represents the numerical values of *f*′′(0) with the previous published results in the limiting case of *γ* = 0 and *Ha* = 0. It is concluded that both the results are in good agreement. It is analyzed that *f*′′(0) shows decreasing behavior for higher values of *M* while opposite behavior is observed for increasing *λ*. Table 3 presents comparison of skin friction coefficient with the previous results in limiting cases of *γ* = 0 and *Ha*^2^ = 0. It is evident that both the results match in good agreement. Skin friction coefficient increases with an increase in *M* and it decreases with *λ*. Therefore small values of *M* and large values of *λ* can be used for the reduction of skin friction coefficient. Table 4 depicts the numerical values of Nusselt number for various parameters. It is concluded that Nusselt number increases for larger curvature parameter *γ*, fluid parameter *M*, Prandtl number *Pr* and conjugate parameter *α* while it decreases with Hartman number *Ha*^2^ and heat generation parameter *δ*. As rate of heat transfer is high for large values of curvature parameter *γ* and fluid parameter *M* so these parameters can be used as coolant factor. Thus it is noted that cylindrical shape devices with large curvature i.e, with small radius have high rate of heat transfer. Table 5 presents the numerical values of skin friction for various parameters. It is concluded that skin friction increases for larger curvature parameter *γ* and Hartman number *Ha*^2^ while it decreases with increase in the values of fluid parameter *M*. Table 6 shows comparison of *f*′′(0) for different methods with previous published works [30, 31]. The results are found in good agreement.

**Table 1:** Convergence of series solutions for different order of approximations when *γ* = 0.1, *M* = 0.3, *λ* = 0.1, *Ha* = 0.2, *K* = 0.2, *K*_*s*_ = 0.2, *Sc* = 0.9, *α* = 0.1, *δ* = 0.1 and *Pr* = 1.2.

Order of approximations	−*f*′′(0)	−*θ*′(0)	Φ′(0)
1	1.0222	0.11494	0.05030
5	1.0061	0.11970	0.05671
10	1.0060	0.12078	0.06591
15	1.0060	0.12108	0.07594
20	1.0060	0.12108	0.07594
25	1.0060	0.12108	0.07594

**Table 2:** Comparison of *f*′′(0) of the present results (in brackets) with the previous published work [3] when *γ* = 0 and *Ha* = 0.

*λ*/*M*	0.0	0.2	0.4	0.6	0.8	1.0
0.0	-1	-0.9131	-0.8452	-0.7906	-0.7454	-0.7071
	(-1)	(-0.91287)	(-0.84516)	(-0.79057)	(-0.74536)	(-0.70711)
0.1	-1	-0.9159	-0.8493	-0.7950	-0.7498	-0.7114
	(-1)	(-0.91590)	(-0.84929)	(-0.79503)	(-0.74979)	(-0.71137)
0.2	-1	-0.9190	-0.8536	-0.7997	-0.7544	-0.7158
	(-1)	(-0.91900)	(-0.85358)	(-0.79968)	(-0.75442)	(-0.71584)
0.3	-1	-0.9222	-0.8580	-0.8045	-0.7593	-0.7205
	(-1)	(-0.92218)	(-0.85804)	(-0.80453)	(-0.75927)	(-0.72048)
0.4	-1	-0.9254	-0.8627	-0.8096	-0.7644	-0.7254
	(-1)	(-0.92543)	(-0.86267)	(-0.80960)	(-0.76436)	(-0.72538)
0.5	-1	-0.9288	-0.8675	-0.8149	-0.7697	-0.7305
	(-1)	(-0.92878)	(-0.86749)	(-0.81493)	(-0.76971)	(-0.73053)
0.6	-1	-0.9322	-0.8725	-0.8205	-0.7754	-0.7360
	(-1)	(-0.93221)	(-0.87252)	(-0.82053)	(-0.77534)	(-0.73598)
0.7	-1	-0.9357	-0.878	-0.8264	-0.7813	-0.7418
	(-1)	(-0.93574)	(-0.87777)	(-0.82643)	(-0.78133)	(-0.74174)
0.8	-1	-0.9394	-0.8833	-0.8327	-0.7877	-0.7479
	(-1)	(-0.93938)	(-0.88327)	(-0.83267)	(-0.78768)	(-0.74788)
0.9	-1	-0.9431	-0.8891	-0.8393	-0.7954	-0.7544
	(-1)	(-0.94312)	(-0.88905)	(-0.83930)	(-0.79446)	(-0.75443)
1.0	-1	-0.9470	-0.8951	-0.8464	-0.8017	-0.7615
	(-1)	(-0.94698)	(-0.89513)	(-0.84637)	(-0.80172)	(-0.76145)

**Table 3:** Comparison of skin friction coefficient $Re_{x}^{1/2}C_{f}$ of the present results (in brackets) with the previous published work [3] when *γ* = 0 and *Ha* = 0.

*λ*/*M*	0.0	0.2	0.4	0.6	0.8	1.0
0.0	-1	-1.0954	-1.1832	-1.2649	-1.3416	-1.4142
	(-1)	(-1.09545)	(-1.18322)	(-1.26491)	(-1.34164)	(-1.41421)
0.1	-1	-1.0940	-1.1808	-1.2620	-1.3384	-1.4107
	(-1)	(-1.09395)	(-1.18084)	(-1.26199)	(-1.33838)	(-1.41073)
0.2	-1	-1.0924	-1.1784	-1.2590	-1.3351	-1.4072
	(-1)	(-1.09245)	(-1.17843)	(-1.25902)	(-1.33506)	(-1.40718)
0.3	-1	-1.0909	-1.1776	-1.2560	-1.3317	-1.4036
	(-1)	(-1.09092)	(-1.17598)	(-1.25600)	(-1.33167)	(-1.40356)
0.4	-1	-1.0894	-1.1735	-1.2529	-1.3282	-1.3999
	(-1)	(-1.08938)	(-1.17349)	(-1.25291)	(-1.32821)	(-1.3999)
0.5	-1	-1.0878	-1.1710	-1.2498	-1.3247	-1.3961
	(-1)	(-1.08782)	(-1.17096)	(-1.24976)	(-1.32467)	(-1.39609)
0.6	-1	-1.0862	-1.1684	-1.2466	-1.3211	-1.3922
	(-1)	(-1.08625)	(-1.16838)	(-1.24655)	(-1.32106)	(-1.39223)
0.7	-1	-1.0847	-1.1658	-1.2433	-1.3174	-1.3883
	(-1)	(-1.08465)	(-1.16575)	(-1.24327)	(-1.31736)	(-1.38827)
0.8	-1	-1.0830	-1.1631	-1.2399	-1.3136	-1.3842
	(-1)	(-1.08304)	(-1.16308)	(-1.23991)	(-1.31357)	(-1.38422)
0.9	-1	-1.0814	-1.1603	-1.2365	-1.3097	-1.3801
	(-1)	(-1.08141)	(-1.16035)	(-1.23646)	(-1.30968)	(-1.38006)
1.0	-1	-1.0798	-1.1576	-1.2329	-1.3057	-1.3758
	(-1)	(-1.07975)	(-1.15756)	(-1.23293)	(-1.30569)	(-1.37578)

**Table 4:** Numerical values of Nusselt number for different parameters.

*α*	*M*	*γ*	*Ha*	*δ*	Pr	$Nu_{z}Re_{z}^{-1/2}$
0.1	0.3	0.2	0.1	0.2	1.2	0.5712
0.3						0.5827
0.5						0.6140
0.1	0.0	0.2	0.1	0.2	1.2	0.3687
	0.1					0.3968
	0.4					0.3990
0.1	0.3	0.3	0.1	0.2	1.2	0.5751
		0.5				0.5841
		0.7				0.6138
0.1	0.3	0.2	0.1	0.2	1.2	0.4208
			0.1			0.3852
			0.3			0.3605
0.1	0.3	0.2	0.1	0.0	1.2	0.4307
				0.1		0.4275
				0.2		0.4119
0.1	0.3	0.2	0.1	0.1	0.8	0.3843
					1	0.4115
					1.2	0.4407

**Table 5:** Numerical values of skin friction for different parameters.

*M*	*Ha*	*γ*	$C_{f}Re_{z}^{1/2}$
0.0	0.1	0.2	0.050318
0.1			0.048337
0.3			0.045014
0.3	0.0	0.2	0.036821
	0.1		0.045014
	0.3		0.052441
0.3	0.1	0.0	0.036719
		0.1	0.041062
		0.2	0.045014

**Table 6:** Comparison of *f*′′(0) when *γ* = 0, *λ* = 0 and *M* = 0. Here PR stands for present results.

		[30]		[31]	
Ha	HPM	MHPM	Exact Solution	Exact Solution	PR
0	1	1	1	1	1
0.5				-1.1180	-1.118034
1	-1.41421	-1.41421	-1.41421		-1.414232
5	-2.44948	-2.44948	-2.44948		-2.449474

## 5 Final remarks

Here the influence of homogeneous-heterogeneous reactions in the flow of Powell-Eyring fluid by a stretching cylinder is discussed in the presence of heat generation/absorption and Newtonian heating. Main points through convergent solutions are as follows:

- The velocity profile increases for larger values of fluid parameter *M* while the temperature and thermal layer thickness decrease. Fluid parameter inversely relates the viscosity of fluid. As a result fluid becomes less viscous as M increases. Thus the temperature profile decreases. This parameter can be used to control the heat transfer in the industrial and engineering processes. The fluid parameter also plays important role in polymer solutions, paint, flow of blood, flow of liquid metals etc.
- The temperature field near the surface of cylinder decreases for lager values of curvature parameter. Further the heat transfer through conduction reduce for higher values of curvature parameter due to reduction of contact area of fluid particles. Thus this parameter can be used as a cooling agents in industrial processes.
- The temperature and thermal boundary layer thickness are enhanced when Hartman number increases. Physically the Lorentz force appeared due to the applied magnetic field which acts as a retarding force and causes the reduction in the flow and enhancement in thermal boundary layer thickness. It has significant applications in industrial manufacturing processes such as plasma studies, petroleum industries, magneto-hydrodynamics power generator, cooling of nuclear reactors, boundary layer control in aerodynamics, glass fiber production and paper production.
- Homogeneous and heterogeneous parameters have opposite behavior for concentration profile. It is observed concentration at the surface decreases as the strength of the heterogeneous reaction increases. It is due to inverse relation with mass diffusivity. Such reactions occur in combustion, catalysis and bio chemical systems.
- Skin friction coefficient directly relates with fluid parameter *M* and inversely relates with the fluid parameter *λ*. Hence smaller values of M and larger values of *λ* can be used for the reduction of skin friction coefficient. Larger values of curvature parameter *λ* and fluid parameter *M* have high rate of heat transfer.

It is hoped that the present investigation serves as impetus for the researchers and scientists to develop catalytic processes involving homogeneous-heterogeneous reactions which can be operated at high temperatures such as reactions occurring in combustion, bio chemical systems etc. This analysis can be extended to variable sheet thickness, melting heat transfer and Cattaneo-Christov heat flux.
